# Supplementary material for: Organoboron Complexes as Thermally Activated Delayed Fluorescence (TADF) Materials for Organic Light-Emitting Diodes (OLEDs): A Computational Study
Source: Molecules. 2023 Oct 6;28(19):6952. doi: 10.3390/molecules28196952 (PMC10574585; doi:10.3390/molecules28196952)
Supplement: Supplementary file 1 [file molecules-28-06952-s001.zip › molecules-2592571-supplementary.pdf]

Organoboron Complexes as Thermally Activated Delayed Fluorescence (TADF) Materials for Organic Light-Emitting Diodes (OLEDs): A computational Study.

Jamilah A. Asiri [1,2], Walid M. I. Hasan [1], Abdesslem Jedidi [1], Shaaban A. Elroby [1,3,*], Saadullah G. Aziz [1] and Osman I. Osman [1,4,*]

[1] Chemistry Department, Faculty of Science, King Abdulaziz University, Jeddah 21589, Saudi Arabia; jmohammedasiri@stu.kau.edu.sa (J.A.A.);
whassan@kau.edu.sa (W.M.I.H.); ajedidi@kau.edu.sa (A.J.); saziz@kau.edu.sa (S.G.A.)
[2] Department of Chemistry, College of Arts and Sciences, Prince Sattam bin Abdulaziz University, Wadi Ad-Dwasir, 62481, Saudi Arabia
[3] Chemistry Department, Faculty of Science, Beni-Suif University, Beni-Suif,62521, Egypt
[4] Chemistry Department, Faculty of Science, University of Khartoum, Khartoum P.O. Box 321, Sudan
* Correspondence: skamel@kau.edu.sa (S.A.E.); oabdelkarim@kau.edu.sa (O.I.O.)

Table of Contents

Abstract ………………………………………………………………...1
Table S1: The dihedral angles of the substrates in degrees…………2
Table S2: Spin-orbit coupling SOC …………………………………2
Table S3: The reorganization energies of the S_1 and T_1 states …..….2
Table S4: IFCT of the second excited triplet state T_2 ……………….3
Table S5: IFCT of the third excited triplet state T_3…………………..3
Table S6: XYZ coordinates of the Ac-B-X complexes ….…………..…4
Figure S1: UV-Vis. spectra of Ac-B-X complexes…………………30
Figure S2: NTO of the second excited triplet state T_2………………32
Figure S2: NTO of the third excited triplet state T_3…………………33
References……………………………………………………………34

Abstract: We report on organoboron complexes characterized by very small energy gaps (ΔE_{ST}) between their singlet and triplet excited states, which allow for highly efficient harvesting of triplet excitons into singlet states for working as thermally activated delayed fluorescence (TADF) devices. Energy gaps ranging between 0.01 and 0.06 eV with dihedral angles of *ca.* 90° were registered. The spin–orbit couplings between the lowest excited S_1 and T_1 states yielded reversed intersystem crossing rate constants (K_{RISC}) of an average of 10^5 s^{-1}. This setup accomplished radiative decay rates of *ca.* 10^6 s^{-1}, indicating highly potent electroluminescent devices, and hence, being suitable for application as organic light-emitting diodes.

Keywords: organoboron; TADF; RISC; NTO; radiative decay rate; DFT

Table S1. The dihedral angles of the substrates in degrees.

Compound	Angle°
Ac-B	79.63
Ac-B-F	81.28
Ac-B-CN	81.16
Ac-B-NO_2	70.52

Table S2. Spin–orbit coupling SOC $|\langle S_1|\hat{H}_{SOC}|T_1\rangle|$.

Compound	$|\langle S_1|\hat{H}_{SOC}|T_1\rangle|$ cm^{-1}
Ac-B	0.38
Ac-B-F	0.66
Ac-B-CN	0.14
Ac-B-NO_2	1.41

Reorganization energies λ:

The reorganization energy is an average of $\lambda(S_1\text{-}T_1)$ and $\lambda(T_1\text{-}S_1)$.

$\lambda(S_1\text{-}T_1)$ = S_1 energy based on optimized T_1 geometry – optimized S_1 energy;

$\lambda(T_1\text{-}S_1)$ = T_1 energy based on optimized S_1 geometry – optimized T_1 energy.

Table S3. The reorganization energies associated with S_1 and T_1 excited states.

Compound	λ eV
Ac-B	5.0×10^{-1}
Ac-B-F	1.4×10^{-1}
Ac-B-CN	5.7×10^{-4}
Ac-B-NO_2	3.0×10^{-3}

Table S4. IFCT of the second excited triplet (T_2) state.

Compound	CT%	LE%
**Ac-B**	**94.55**	**05.45**
**Ac-B-F**	**20.00**	**80.00**
**Ac-B-CN**	**93.26**	**06.38**
**Ac-B-NO_2**	**92.04**	**07.96**

Table S5. IFCT of the third excited triplet (T_3) state.

Compound	CT%	LE%
**Ac-B**	**47.15**	**52.85**
**Ac-B-F**	**44.06**	**55.94**
**Ac-B-CN**	**23.01**	**76.99**
**Ac-B-NO_2**	**26.52**	**73.48**

Table S6. XYZ Coordinates of the ground S_0 and vertical and adiabatic first excited S_1 and T_1 states of the studied complexes.

Ac-B

(S$_0$)

```
C 3.84893500 -1.99047900 3.04563800
C 3.15314600 -1.32978100 2.03919500
C 3.84245400 -0.74863800 0.96597900
C 5.24378200 -0.87252300 0.87799100
C 5.91545200 -1.51558300 1.91848000
C 5.23667900 -2.07099500 3.00102800
C 5.24767000 0.87253100 -0.88070000
C 3.84644400 0.96477100 -0.75940200
C 3.16057000 2.03658100 -1.34707300
H 2.08171100 2.09421600 -1.27319800
C 3.85946400 3.03672600 -2.01394600
C 5.24711400 2.98735000 -2.09364700
```

C 5.92261300 1.90681900 -1.53033300
H 3.29889800 -2.43080800 3.87100300
H 2.07418000 -1.25519100 2.09387100
H 6.99469600 -1.60696100 1.87989800
H 5.78750100 -2.57227500 3.78953300
H 3.31214000 3.86087900 -2.45990700
H 5.80050000 3.77069300 -2.60017700
H 7.00181300 1.86470000 -1.62070500
N 3.15929200 -0.03420100 -0.03961400
C 5.93399400 -0.40736600 -0.40411100
C 5.70930000 -1.49846300 -1.48708800
H 6.16033700 -2.44379200 -1.16903000
H 4.64345900 -1.66862000 -1.65840800
H 6.16342400 -1.18924300 -2.43385700
C 7.44611600 -0.22290000 -0.21734900
H 7.92009600 0.06531700 -1.15873200
H 7.67286600 0.53727900 0.53564900
H 7.91599800 -1.16267400 0.08269600
C 1.03086400 -0.86533100 -0.88279100
C 1.72847600 -0.01588200 -0.02503200
C 1.02955400 0.83803700 0.83431900
C -0.36046600 0.84251000 0.82055900
C -1.09001800 -0.01598900 -0.02348700
C -0.36056500 -0.87108000 -0.86839900
H 1.59065800 -1.51809300 -1.54393300
H 1.58762300 1.49219100 1.49631500
H -0.89958700 1.51978300 1.47712500
H -0.89905400 -1.54915400 -1.52452500
B -2.66100100 -0.02141400 -0.02197600
C -5.05290100 -2.45680400 -1.88347200
C -4.39676100 -1.28923200 -1.49554200
C -3.42068200 -1.33197300 -0.46765100
C -3.15588800 -2.57514000 0.15241500
C -3.85523500 -3.71871800 -0.24481500
C -4.80035900 -3.68448200 -1.26722000
H -5.79174100 -2.40820900 -2.68040600
H -3.65577500 -4.66107200 0.26104500
C -3.43442200 1.28123500 0.42298300
C -4.41612400 1.22752000 1.44524900
C -5.08812200 2.38739600 1.82890200
C -4.84671400 3.61781900 1.21362600
C -3.89581300 3.66291700 0.19711200
C -3.18069100 2.52749800 -0.19558300
H -5.83112700 2.33026500 2.62137900
H -3.70459200 4.60736000 -0.30804400
C -4.74404500 0.00016100 -2.20803300
H -5.18234000 0.72939400 -1.52145400
H -3.86077100 0.46971600 -2.65282500
H -5.45940200 -0.18134000 -3.01401500
C -4.75413900 -0.06553300 2.15546700
H -5.18549400 -0.79718800 1.46709100
H -3.86818700 -0.52887100 2.60120100
H -5.47248100 0.10918000 2.96030300
C -5.52181600 -4.93347400 -1.70922800
H -5.07701900 -5.33824500 -2.62532100
H -5.47327900 -5.71434600 -0.94604400
H -6.57571800 -4.73077400 -1.92104500
C -2.14627400 -2.73096700 1.27295800
H -1.16351700 -3.01287600 0.88020700
H -2.00314900 -1.81359100 1.84732000
H -2.46216800 -3.51388300 1.96800800
C -5.58610700 4.85819400 1.65013400
H -5.15037900 5.27028800 2.56733100
H -5.54418500 5.63821200 0.88569800
H -6.63825300 4.64257900 1.85787000
C -2.16709400 2.69576600 -1.31070400
H -1.19195600 2.99568600 -0.91201600
H -2.00495800 1.77939800 -1.88130200
H -2.49114600 3.47156500 -2.00999000

Vertical (S_1)

```
C 3.84894 -1.99048 3.04564
C 3.15315 -1.32978 2.03919
C 3.84245 -0.74864 0.96598
C 5.24378 -0.87252 0.87799
C 5.91545 -1.51558 1.91848
C 5.23668 -2.07099 3.00103
C 5.24767 0.87253 -0.8807
C 3.84644 0.96477 -0.7594
C 3.16057 2.03658 -1.34707
H 2.08171 2.09422 -1.2732
C 3.85946 3.03673 -2.01395
C 5.24711 2.98735 -2.09365
C 5.92261 1.90682 -1.53033
H 3.2989 -2.43081 3.871
H 2.07418 -1.25519 2.09387
H 6.9947 -1.60696 1.8799
H 5.7875 -2.57228 3.78953
H 3.31214 3.86088 -2.45991
H 5.8005 3.77069 -2.60018
H 7.00181 1.8647 -1.62071
N 3.15929 -0.0342 -0.03961
C 5.93399 -0.40737 -0.40411
C 5.7093 -1.49846 -1.48709
H 6.16034 -2.44379 -1.16903
H 4.64346 -1.66862 -1.65841
H 6.16342 -1.18924 -2.43386
C 7.44612 -0.2229 -0.21735
H 7.9201 0.06532 -1.15873
H 7.67287 0.53728 0.53565
H 7.916 -1.16267 0.0827
C 1.03086 -0.86533 -0.88279
C 1.72848 -0.01588 -0.02503
C 1.02955 0.83804 0.83432
C -0.36047 0.84251 0.82056
C -1.09002 -0.01599 -0.02349
C -0.36057 -0.87108 -0.8684
H 1.59066 -1.51809 -1.54393
H 1.58762 1.49219 1.49632
H -0.89959 1.51978 1.47713
H -0.89905 -1.54915 -1.52453
B -2.661 -0.02141 -0.02198
C -5.0529 -2.4568 -1.88347
C -4.39676 -1.28923 -1.49554
C -3.42068 -1.33197 -0.46765
C -3.15589 -2.57514 0.15242
C -3.85524 -3.71872 -0.24482
C -4.80036 -3.68448 -1.26722
H -5.79174 -2.40821 -2.68041
H -3.65578 -4.66107 0.26105
C -3.43442 1.28124 0.42298
C -4.41612 1.22752 1.44525
C -5.08812 2.3874 1.8289
C -4.84671 3.61782 1.21363
C -3.89581 3.66292 0.19711
C -3.18069 2.5275 -0.19558
H -5.83113 2.33027 2.62138
H -3.70459 4.60736 -0.30804
C -4.74405 0.00016 -2.20803
H -5.18234 0.72939 -1.52145
H -3.86077 0.46972 -2.65283
H -5.4594 -0.18134 -3.01402
C -4.75414 -0.06553 2.15547
H -5.18549 -0.79719 1.46709
H -3.86819 -0.52887 2.6012
H -5.47248 0.10918 2.9603
C -5.52182 -4.93347 -1.70923
```

H -5.07702 -5.33825 -2.62532
H -5.47328 -5.71435 -0.94604
H -6.57572 -4.73077 -1.92105
C -2.14627 -2.73097 1.27296
H -1.16352 -3.01288 0.88021
H -2.00315 -1.81359 1.84732
H -2.46217 -3.51388 1.96801
C -5.58611 4.85819 1.65013
H -5.15038 5.27029 2.56733
H -5.54419 5.63821 0.8857
H -6.63825 4.64258 1.85787
C -2.16709 2.69577 -1.3107
H -1.19196 2.99569 -0.91202
H -2.00496 1.7794 -1.8813
H -2.49115 3.47157 -2.00999

Vertical (T_1)

C 3.84894 -1.99048 3.04564
C 3.15315 -1.32978 2.03919
C 3.84245 -0.74864 0.96598
C 5.24378 -0.87252 0.87799
C 5.91545 -1.51558 1.91848
C 5.23668 -2.07099 3.00103
C 5.24767 0.87253 -0.8807
C 3.84644 0.96477 -0.7594
C 3.16057 2.03658 -1.34707
H 2.08171 2.09422 -1.2732
C 3.85946 3.03673 -2.01395
C 5.24711 2.98735 -2.09365
C 5.92261 1.90682 -1.53033
H 3.2989 -2.43081 3.871
H 2.07418 -1.25519 2.09387
H 6.9947 -1.60696 1.8799
H 5.7875 -2.57228 3.78953
H 3.31214 3.86088 -2.45991
H 5.8005 3.77069 -2.60018
H 7.00181 1.8647 -1.62071
N 3.15929 -0.0342 -0.03961
C 5.93399 -0.40737 -0.40411
C 5.7093 -1.49846 -1.48709
H 6.16034 -2.44379 -1.16903
H 4.64346 -1.66862 -1.65841
H 6.16342 -1.18924 -2.43386
C 7.44612 -0.2229 -0.21735
H 7.9201 0.06532 -1.15873
H 7.67287 0.53728 0.53565
H 7.916 -1.16267 0.0827
C 1.03086 -0.86533 -0.88279
C 1.72848 -0.01588 -0.02503
C 1.02955 0.83804 0.83432
C -0.36047 0.84251 0.82056
C -1.09002 -0.01599 -0.02349
C -0.36057 -0.87108 -0.8684
H 1.59066 -1.51809 -1.54393
H 1.58762 1.49219 1.49632
H -0.89959 1.51978 1.47713
H -0.89905 -1.54915 -1.52453
B -2.661 -0.02141 -0.02198
C -5.0529 -2.4568 -1.88347
C -4.39676 -1.28923 -1.49554
C -3.42068 -1.33197 -0.46765
C -3.15589 -2.57514 0.15242
C -3.85524 -3.71872 -0.24482
C -4.80036 -3.68448 -1.26722
H -5.79174 -2.40821 -2.68041
H -3.65578 -4.66107 0.26105
C -3.43442 1.28124 0.42298
C -4.41612 1.22752 1.44525
C -5.08812 2.3874 1.8289

C -4.84671 3.61782 1.21363
C -3.89581 3.66292 0.19711
C -3.18069 2.5275 -0.19558
H -5.83113 2.33027 2.62138
H -3.70459 4.60736 -0.30804
C -4.74405 0.00016 -2.20803
H -5.18234 0.72939 -1.52145
H -3.86077 0.46972 -2.65283
H -5.4594 -0.18134 -3.01402
C -4.75414 -0.06553 2.15547
H -5.18549 -0.79719 1.46709
H -3.86819 -0.52887 2.6012
H -5.47248 0.10918 2.9603
C -5.52182 -4.93347 -1.70923
H -5.07702 -5.33825 -2.62532
H -5.47328 -5.71435 -0.94604
H -6.57572 -4.73077 -1.92105
C -2.14627 -2.73097 1.27296
H -1.16352 -3.01288 0.88021
H -2.00315 -1.81359 1.84732
H -2.46217 -3.51388 1.96801
C -5.58611 4.85819 1.65013
H -5.15038 5.27029 2.56733
H -5.54419 5.63821 0.8857
H -6.63825 4.64258 1.85787
C -2.16709 2.69577 -1.3107
H -1.19196 2.99569 -0.91202
H -2.00496 1.7794 -1.8813
H -2.49115 3.47157 -2.00999

Adiabatic S_1

C 3.83896200 -1.79616300 3.16882300
C 3.16904700 -1.17444300 2.13355500
C 3.89412900 -0.58332200 1.07148300
C 5.31020400 -0.62392000 1.05672100
C 5.94787700 -1.26478100 2.11610700
C 5.23452700 -1.84476000 3.16302700
C 5.24196300 0.66381900 -1.10415300
C 3.82872000 0.66134100 -1.00628400
C 3.03811700 1.30136600 -1.99092500
H 1.96309600 1.28377800 -1.88444100
C 3.64146100 1.93312600 -3.06027500
C 5.03346300 1.93515200 -3.17082300
C 5.81129300 1.30585100 -2.20098400
H 3.27679400 -2.24436800 3.97917800
H 2.08989900 -1.12595500 2.11477400
H 7.03080000 -1.32114400 2.13081000
H 5.76814000 -2.33321400 3.97058000
H 3.02937200 2.42209200 -3.80850700
H 5.51487400 2.42393700 -4.01034200
H 6.89008700 1.31843100 -2.31119500
N 3.18343100 0.03240400 0.04996200
C 6.12877700 -0.02032700 -0.07471200
C 6.91340400 -1.16047800 -0.77834600
H 7.57861400 -1.66402600 -0.07273000
H 6.22695900 -1.90251400 -1.19312000
H 7.52464500 -0.76368200 -1.59253700
C 7.11830800 1.01961400 0.51175800
H 7.73925400 1.45161200 -0.27620000
H 6.57977400 1.83024800 1.00829900
H 7.78708300 0.55418600 1.23925900
C 1.05260000 -1.04553700 -0.57306200
C 1.74463400 0.00770600 0.07681200
C 1.02787700 1.05636500 0.70748800
C -0.34498800 1.03528900 0.68319400
C -1.10605300 -0.00545900 0.04180700
C -0.32010100 -1.03974900 -0.57940800

H 1.61129600 -1.83597100 -1.06642700
H 1.56709100 1.85587000 1.20796400
H -0.88625300 1.83255300 1.18552800
H -0.84110300 -1.84338300 -1.09273200
B -2.62346800 -0.01107100 0.01825500
C -5.04349700 -2.40559100 -1.92743400
C -4.34095600 -1.26633600 -1.53415800
C -3.41686100 -1.31747600 -0.45847100
C -3.25156400 -2.56448200 0.19117500
C -3.98036300 -3.68735900 -0.22153700
C -4.87936500 -3.63292400 -1.28164400
H -5.74490900 -2.33485700 -2.75721700
H -3.84190900 -4.62919800 0.30735500
C -3.44221100 1.28896400 0.46837500
C -4.40184400 1.23003500 1.51191400
C -5.12695100 2.36344500 1.88064700
C -4.95140400 3.59196200 1.24038000
C -4.01756900 3.65394300 0.21116100
C -3.26584000 2.53725400 -0.17610100
H -5.85557100 2.28684200 2.68604900
H -3.86984800 4.59672500 -0.31353800
C -4.58095900 0.01772500 -2.29593600
H -4.97092900 0.80360100 -1.64337000
H -3.65177900 0.39968000 -2.73187700
H -5.29351000 -0.13516300 -3.11172400
C -4.65790200 -0.05623900 2.26465400
H -5.02803800 -0.84163000 1.59998500
H -3.73956500 -0.43648800 2.72408500
H -5.39241600 0.09267600 3.06147900
C -5.64310400 -4.85542000 -1.73070200
H -5.22122600 -5.27098200 -2.65349900
H -5.61544900 -5.64306100 -0.97248500
H -6.69293400 -4.61975400 -1.93250100
C -2.30104500 -2.75051200 1.35600400
H -1.31538000 -3.08513000 1.01072300
H -2.13542800 -1.81925700 1.90016000
H -2.68175800 -3.50303600 2.05389500
C -5.73831400 4.80885200 1.66398900
H -5.33315000 5.24238300 2.58604200
H -5.71274400 5.58719900 0.89612200
H -6.78687400 4.56067000 1.85667500
C -2.27964600 2.73080700 -1.30984100
H -1.30764800 3.07135500 -0.93298800
H -2.09191500 1.80135300 -1.85002000
H -2.64308100 3.48211900 -2.01817400

Adiabatic T_1

C 3.63299700 -1.70229400 3.24280800
C 2.99293500 -1.12292600 2.15298100
C 3.73433700 -0.69027600 1.04462000
C 5.13039600 -0.88321100 1.01151100
C 5.74662100 -1.43983600 2.13298000
C 5.01647200 -1.84627000 3.24796000
C 5.25577400 0.66539900 -0.91698600
C 3.85732200 0.82956200 -0.84614800
C 3.23657400 1.86290500 -1.56190300
H 2.15977500 1.97236700 -1.52712300
C 3.99751300 2.75662200 -2.30729400
C 5.38312100 2.64019100 -2.34022400
C 5.99405400 1.59566700 -1.64947900
H 3.04272000 -2.02838600 4.09319300
H 1.91741700 -0.99684200 2.16557700
H 6.82140600 -1.57998900 2.13684500
H 5.52384400 -2.28456900 4.10056900
H 3.49941000 3.55277100 -2.85145400
H 5.98478500 3.34033100 -2.90969900
H 7.07206200 1.49701000 -1.70329400
N 3.10777500 -0.05594400 -0.04699500

C 5.86939600 -0.58788900 -0.29323000
C 5.61240500 -1.77437100 -1.26278300
H 6.01248400 -2.70076000 -0.83834800
H 4.54281300 -1.91332400 -1.43898100
H 6.09698800 -1.58685900 -2.22631900
C 7.38473800 -0.45518800 -0.08969700
H 7.88940800 -0.29101400 -1.04496200
H 7.63376100 0.36973100 0.58412000
H 7.80258700 -1.37792800 0.32045700
C 0.96599100 -0.92168900 -0.82859700
C 1.67813500 0.01319200 -0.07824600
C 0.98780500 0.99422800 0.64192100
C -0.40026100 1.03314600 0.60070000
C -1.15213900 0.09591400 -0.14103900
C -0.42457700 -0.88285900 -0.84797300
H 1.51328600 -1.67673700 -1.38329400
H 1.55126600 1.71234100 1.22929600
H -0.92042900 1.80290100 1.16559800
H -0.96860300 -1.63133500 -1.41695000
B -2.72258500 0.06815000 -0.11814100
C -4.88629900 -2.70788000 -1.80456800
C -4.27786300 -1.47808300 -1.54908900
C -3.44085500 -1.31836300 -0.42097500
C -3.24022800 -2.43142200 0.42514200
C -3.87292700 -3.64683200 0.14623700
C -4.69723700 -3.80853700 -0.96573400
H -5.52613000 -2.81202000 -2.67867400
H -3.72239500 -4.48660100 0.82156600
C -3.54142100 1.35441000 0.15422600
C -4.76638900 1.40122600 0.96460000
C -5.06752500 2.57209500 1.59375500
C -4.33194600 3.80541000 1.37535600
C -3.37253500 3.82540700 0.35116100
C -3.05647900 2.68265400 -0.35344000
H -5.89495600 2.59782900 2.29931000
H -2.93694800 4.77698800 0.05397400
C -4.51402100 -0.33348500 -2.50971600
H -4.89534000 0.55588100 -1.99505000
H -3.58372600 -0.04081600 -3.00976200
H -5.23547300 -0.60648500 -3.28412700
C -5.57765900 0.16613800 1.24265000
H -5.95852100 -0.28347600 0.32157600
H -4.97162200 -0.60442900 1.72877300
H -6.42506500 0.40302000 1.89082700
C -5.36358600 -5.12860500 -1.26781800
H -4.93638900 -5.58812300 -2.16597700
H -5.24366000 -5.83693300 -0.44414800
H -6.43543700 -4.99947900 -1.44875700
C -2.36484700 -2.33690800 1.65606900
H -1.30379000 -2.40105400 1.39237000
H -2.50105500 -1.38515800 2.17948200
H -2.58558800 -3.14465400 2.35905000
C -4.70520200 5.02599900 2.15461500
H -4.51956100 4.87729600 3.22648200
H -4.13999600 5.90333600 1.83261400
H -5.77519000 5.24829600 2.05302900
C -2.21521500 2.74857100 -1.59070800
H -1.16415200 2.51188300 -1.38148400
H -2.54946500 2.01091100 -2.32816100
H -2.25628500 3.74406100 -2.04035800

Ac-B-F

S_0

C 3.76000800 -1.81613400 3.16009500
C 3.06269800 -1.16132800 2.15102100
C 3.74263700 -0.65695700 1.03642600
C 5.13199400 -0.84505100 0.90774900

C 5.80879600 -1.47809100 1.95137900
C 5.14066800 -1.96123400 3.07434500
C 5.15467500 0.83367800 -0.91653900
C 3.76572300 0.99605900 -0.75449600
C 3.10706500 2.07807400 -1.34982700
H 2.03527400 2.18936900 -1.23872100
C 3.82450800 3.02158400 -2.07664200
C 5.20463200 2.90325500 -2.20308900
C 5.85192700 1.81352000 -1.62455100
H 3.21895200 -2.19842200 4.01941500
H 1.99026200 -1.03634700 2.23767100
H 6.88105200 -1.61954600 1.88321600
H 5.69383200 -2.45727300 3.86448300
H 3.30010000 3.85628300 -2.53010500
H 5.77432000 3.64144800 -2.75702000
H 6.92410800 1.71923800 -1.75017100
N 3.06225500 0.05345400 0.02464700
C 5.79582700 -0.46021500 -0.41471900
C 5.47738500 -1.57810500 -1.44596600
H 5.89279200 -2.53174500 -1.10458700
H 4.40005900 -1.69984500 -1.57643700
H 5.91606000 -1.32944800 -2.41760200
C 7.32060300 -0.34201200 -0.28725400
H 7.77227700 -0.10779200 -1.25423200
H 7.61077100 0.43015600 0.43096500
H 7.75690200 -1.29311100 0.02729800
C 0.93967200 -0.80868500 -0.82250500
C 1.64792900 0.05986400 0.01266100
C 0.88817200 0.91317100 0.82193900
C -0.49688800 0.91371900 0.80457700
C -1.18733600 0.01766800 -0.02864900
C -0.44552800 -0.85069000 -0.84478000
H -1.03474200 1.60952800 1.43932100
H -0.94195800 -1.56463100 -1.49288500
B -2.76473100 -0.02243300 -0.03704300
C -5.07271800 -2.63820900 -1.74083100
C -4.46159300 -1.42736000 -1.41875500
C -3.47994300 -1.37923600 -0.39568700
C -3.16597600 -2.57630800 0.28935100
C -3.82055100 -3.76542300 -0.04506300
C -4.76885500 -3.82163200 -1.06348900
H -5.81510500 -2.66025200 -2.53546400
H -3.58236400 -4.67155200 0.50787200
C -3.55919100 1.28977700 0.32038400
C -4.55515600 1.27550500 1.33105500
C -5.23513600 2.44877300 1.65470600
C -4.98865600 3.65248700 0.99031300
C -4.02733800 3.65652700 -0.01749800
C -3.30420000 2.50799100 -0.35202800
H -5.98696900 2.42417000 2.44026800
H -3.83307600 4.57888200 -0.56059900
C -4.86403500 -0.19176900 -2.19581300
H -5.35156800 0.54421900 -1.55069700
H -4.00136300 0.30368800 -2.65240200
H -5.55693900 -0.44971900 -3.00040300
C -4.90236400 0.01404200 2.09283400
H -5.33980300 -0.74124300 1.43446100
H -4.02154200 -0.43943100 2.55794000
H -5.61980600 0.22783100 2.88880000
C -5.44243100 -5.11801000 -1.43890200
H -5.00082900 -5.53694700 -2.35001000
H -5.34174600 -5.86628600 -0.64875700
H -6.50866700 -4.96919700 -1.63275300
C -2.15552200 -2.63406600 1.41838300
H -1.16383500 -2.91113100 1.04507800
H -2.04243300 -1.68123400 1.93960600
H -2.45112400 -3.38374100 2.15730200
C -5.73432200 4.90805300 1.36818600
H -5.31618200 5.34889900 2.28005400
H -5.67489800 5.66241200 0.57967600

H -6.79079200 4.70082600 1.56169900
C -2.28257300 2.63018300 -1.46579000
H -1.31124700 2.95052800 -1.07389000
H -2.11629900 1.69122800 -1.99762900
H -2.60368400 3.37480700 -2.19918200
F 1.54163700 1.75700600 1.63031100
F 1.63396700 -1.63557400 -1.61409800

Vertical S_1 :

C 3.76001 -1.81613 3.1601
C 3.0627 -1.16133 2.15102
C 3.74264 -0.65696 1.03643
C 5.13199 -0.84505 0.90775
C 5.8088 -1.47809 1.95138
C 5.14067 -1.96123 3.07435
C 5.15468 0.83368 -0.91654
C 3.76572 0.99606 -0.7545
C 3.10706 2.07807 -1.34983
H 2.03527 2.18937 -1.23872
C 3.82451 3.02158 -2.07664
C 5.20463 2.90326 -2.20309
C 5.85193 1.81352 -1.62455
H 3.21895 -2.19842 4.01942
H 1.99026 -1.03635 2.23767
H 6.88105 -1.61955 1.88322
H 5.69383 -2.45727 3.86448
H 3.3001 3.85628 -2.53011
H 5.77432 3.64145 -2.75702
H 6.92411 1.71924 -1.75017
N 3.06225 0.05345 0.02465
C 5.79583 -0.46022 -0.41472
C 5.47738 -1.57811 -1.44597
H 5.89279 -2.53175 -1.10459
H 4.40006 -1.69985 -1.57644
H 5.91606 -1.32945 -2.4176
C 7.3206 -0.34201 -0.28725
H 7.77228 -0.10779 -1.25423
H 7.61077 0.43016 0.43097
H 7.7569 -1.29311 0.0273
C 0.93967 -0.80869 -0.82251
C 1.64793 0.05986 0.01266
C 0.88817 0.91317 0.82194
C -0.49689 0.91372 0.80458
C -1.18734 0.01767 -0.02865
C -0.44553 -0.85069 -0.84478
H -1.03474 1.60953 1.43932
H -0.94196 -1.56463 -1.49289
B -2.76473 -0.02243 -0.03704
C -5.07272 -2.63821 -1.74083
C -4.46159 -1.42736 -1.41876
C -3.47994 -1.37924 -0.39569
C -3.16598 -2.57631 0.28935
C -3.82055 -3.76542 -0.04506
C -4.76886 -3.82163 -1.06349
H -5.81511 -2.66025 -2.53546
H -3.58236 -4.67155 0.50787
C -3.55919 1.28978 0.32038
C -4.55516 1.27551 1.33106
C -5.23514 2.44877 1.65471
C -4.98866 3.65249 0.99031
C -4.02734 3.65653 -0.0175
C -3.3042 2.50799 -0.35203
H -5.98697 2.42417 2.44027
H -3.83308 4.57888 -0.5606
C -4.86404 -0.19177 -2.19581
H -5.35157 0.54422 -1.5507
H -4.00136 0.30369 -2.6524

H -5.55694 -0.44972 -3.0004
C -4.90236 0.01404 2.09283
H -5.3398 -0.74124 1.43446
H -4.02154 -0.43943 2.55794
H -5.61981 0.22783 2.8888
C -5.44243 -5.11801 -1.4389
H -5.00083 -5.53695 -2.35001
H -5.34175 -5.86629 -0.64876
H -6.50867 -4.9692 -1.63275
C -2.15552 -2.63407 1.41838
H -1.16384 -2.91113 1.04508
H -2.04243 -1.68123 1.93961
H -2.45112 -3.38374 2.1573
C -5.73432 4.90805 1.36819
H -5.31618 5.3489 2.28005
H -5.6749 5.66241 0.57968
H -6.79079 4.70083 1.5617
C -2.28257 2.63018 -1.46579
H -1.31125 2.95053 -1.07389
H -2.1163 1.69123 -1.99763
H -2.60368 3.37481 -2.19918
F 1.54164 1.75701 1.63031
F 1.63397 -1.63557 -1.6141

Vertical T_1

C 3.76001 -1.81613 3.1601
C 3.0627 -1.16133 2.15102
C 3.74264 -0.65696 1.03643
C 5.13199 -0.84505 0.90775
C 5.8088 -1.47809 1.95138
C 5.14067 -1.96123 3.07435
C 5.15468 0.83368 -0.91654
C 3.76572 0.99606 -0.7545
C 3.10706 2.07807 -1.34983
H 2.03527 2.18937 -1.23872
C 3.82451 3.02158 -2.07664
C 5.20463 2.90326 -2.20309
C 5.85193 1.81352 -1.62455
H 3.21895 -2.19842 4.01942
H 1.99026 -1.03635 2.23767
H 6.88105 -1.61955 1.88322
H 5.69383 -2.45727 3.86448
H 3.3001 3.85628 -2.53011
H 5.77432 3.64145 -2.75702
H 6.92411 1.71924 -1.75017
N 3.06225 0.05345 0.02465
C 5.79583 -0.46022 -0.41472
C 5.47738 -1.57811 -1.44597
H 5.89279 -2.53175 -1.10459
H 4.40006 -1.69985 -1.57644
H 5.91606 -1.32945 -2.4176
C 7.3206 -0.34201 -0.28725
H 7.77228 -0.10779 -1.25423
H 7.61077 0.43016 0.43097
H 7.7569 -1.29311 0.0273
C 0.93967 -0.80869 -0.82251
C 1.64793 0.05986 0.01266
C 0.88817 0.91317 0.82194
C -0.49689 0.91372 0.80458
C -1.18734 0.01767 -0.02865
C -0.44553 -0.85069 -0.84478
H -1.03474 1.60953 1.43932
H -0.94196 -1.56463 -1.49289
B -2.76473 -0.02243 -0.03704
C -5.07272 -2.63821 -1.74083
C -4.46159 -1.42736 -1.41876
C -3.47994 -1.37924 -0.39569
C -3.16598 -2.57631 0.28935
C -3.82055 -3.76542 -0.04506

C -4.76886 -3.82163 -1.06349
H -5.81511 -2.66025 -2.53546
H -3.58236 -4.67155 0.50787
C -3.55919 1.28978 0.32038
C -4.55516 1.27551 1.33106
C -5.23514 2.44877 1.65471
C -4.98866 3.65249 0.99031
C -4.02734 3.65653 -0.0175
C -3.3042 2.50799 -0.35203
H -5.98697 2.42417 2.44027
H -3.83308 4.57888 -0.5606
C -4.86404 -0.19177 -2.19581
H -5.35157 0.54422 -1.5507
H -4.00136 0.30369 -2.6524
H -5.55694 -0.44972 -3.0004
C -4.90236 0.01404 2.09283
H -5.3398 -0.74124 1.43446
H -4.02154 -0.43943 2.55794
H -5.61981 0.22783 2.8888
C -5.44243 -5.11801 -1.4389
H -5.00083 -5.53695 -2.35001
H -5.34175 -5.86629 -0.64876
H -6.50867 -4.9692 -1.63275
C -2.15552 -2.63407 1.41838
H -1.16384 -2.91113 1.04508
H -2.04243 -1.68123 1.93961
H -2.45112 -3.38374 2.1573
C -5.73432 4.90805 1.36819
H -5.31618 5.3489 2.28005
H -5.6749 5.66241 0.57968
H -6.79079 4.70083 1.5617
C -2.28257 2.63018 -1.46579
H -1.31125 2.95053 -1.07389
H -2.1163 1.69123 -1.99763
H -2.60368 3.37481 -2.19918
F 1.54164 1.75701 1.63031
F 1.63397 -1.63557 -1.6141

Adiabatic S_1

C 4.36800800 -1.71398800 3.20043200
C 3.49182700 -1.23031500 2.24982700
C 3.98374700 -0.61086300 1.07745200
C 5.37745200 -0.48201000 0.86422000
C 6.22956900 -0.98045000 1.84688500
C 5.74488500 -1.58849000 3.00243700
C 4.85615800 0.64104600 -1.32529600
C 3.47965300 0.47071000 -1.03209500
C 2.49228200 0.92135100 -1.94287500
H 1.44789300 0.78187400 -1.70748700
C 2.86454400 1.53294900 -3.12300400
C 4.21772100 1.70936400 -3.41842800
C 5.18941700 1.26495800 -2.52422100
H 3.98123000 -2.18688900 4.09502800
H 2.42277900 -1.31522300 2.37841800
H 7.30212000 -0.89356400 1.71439500
H 6.44168700 -1.96237800 3.74405700
H 2.10182200 1.87296400 -3.81269900
H 4.51824300 2.19109000 -4.34189000
H 6.23394600 1.41409400 -2.77431800
N 3.07210100 -0.13451400 0.14728600
C 5.95221700 0.17354500 -0.38093200
C 6.84386700 -0.85785000 -1.12243500
H 7.65882000 -1.19864800 -0.47959500
H 6.25747800 -1.72833000 -1.42592200
H 7.28885700 -0.41367400 -2.01570900
C 6.80035900 1.40179400 0.04533400
H 7.24974700 1.88264300 -0.82655800

H 6.18103200 2.13692500 0.56440000
H 7.60984200 1.10009700 0.71395000
C 0.91002200 -1.29477600 -0.18309100
C 1.66917300 -0.25833200 0.42107600
C 0.94185200 0.86103000 0.90397400
C -0.41497400 0.93371400 0.83440100
C -1.19290300 -0.11828400 0.22728800
C -0.44821800 -1.25077600 -0.26357700
H -0.91445100 1.79360400 1.26768700
H -0.97084500 -2.08434300 -0.72044000
B -2.70559100 -0.03649300 0.11086000
C -5.10985700 -2.44214000 -1.83138500
C -4.36858100 -1.31684800 -1.47319600
C -3.54478700 -1.32791200 -0.31768300
C -3.51398900 -2.51904800 0.44447100
C -4.27955700 -3.62897000 0.06263800
C -5.08208900 -3.61518700 -1.07275800
H -5.72882600 -2.40503700 -2.72632800
H -4.24645800 -4.52708400 0.67752400
C -3.45158300 1.34442900 0.41643900
C -4.45641800 1.43394000 1.41384900
C -5.09962900 2.64470300 1.67060000
C -4.79638100 3.80729400 0.95868400
C -3.82175000 3.72133100 -0.02982500
C -3.14909500 2.52376300 -0.30511200
H -5.86265600 2.68333500 2.44594700
H -3.57576200 4.60916800 -0.61038500
C -4.43974100 -0.09854900 -2.36617200
H -4.75049100 0.79087900 -1.81169700
H -3.46117900 0.12374400 -2.80587200
H -5.14444200 -0.25276300 -3.18825200
C -4.84729400 0.22900600 2.24009300
H -5.24459200 -0.57419000 1.61333000
H -3.98497900 -0.18228900 2.77454100
H -5.60507500 0.49122500 2.98353400
C -5.89004300 -4.82239600 -1.48407400
H -5.49459200 -5.26892700 -2.40357900
H -5.87715200 -5.59375700 -0.70934400
H -6.93480900 -4.55742200 -1.67696900
C -2.67707000 -2.66340900 1.69936800
H -1.73219700 -3.17644100 1.48455600
H -2.41481700 -1.69879400 2.13590800
H -3.20782500 -3.25427000 2.45270600
C -5.49437700 5.10923500 1.26949400
H -5.09274700 5.56400700 2.18258900
H -5.37139800 5.83386900 0.45972100
H -6.56675900 4.95839800 1.42789500
C -2.11014900 2.55872700 -1.40781600
H -1.13980300 2.89464700 -1.02300900
H -1.94935100 1.57299800 -1.84795400
H -2.41060900 3.25185100 -2.19982600
F 1.67896400 1.85798200 1.45856300
F 1.61703300 -2.33832600 -0.68785000

Adiabatic T_1

C 4.63974600 1.49348100 3.25747500
C 3.67355100 1.07644100 2.35743500
C 4.05509300 0.51613000 1.12690600
C 5.41616400 0.27165000 0.83550800
C 6.36204900 0.70195700 1.76431700
C 5.98944600 1.32613700 2.95377300
C 4.76994400 -0.40636100 -1.47131300
C 3.43246700 -0.09043800 -1.13784600
C 2.44868600 0.03098200 -2.14178900
H 1.44381300 0.32842200 -1.87525800
C 2.77986500 -0.19618500 -3.46682300
C 4.09210600 -0.51836300 -3.80659300
C 5.06951400 -0.61375000 -2.81376400

H 4.33776000 1.94404100 4.19574400
H 2.62577100 1.18955000 2.59163200
H 7.41489000 0.53459600 1.57562200
H 6.75233800 1.65364500 3.65120400
H 2.01906100 -0.10039200 -4.23243200
H 4.36256900 -0.68586400 -4.84315800
H 6.08290400 -0.85827700 -3.10646700
N 3.07387700 0.16140200 0.19010700
C 5.78375600 -0.60954600 -0.35547200
C 5.64962200 -2.08869200 0.12028200
H 6.33001600 -2.27646500 0.95568200
H 4.62946600 -2.30626000 0.44442200
H 5.90049800 -2.76661400 -0.70073300
C 7.22446300 -0.37146200 -0.83489000
H 7.48138800 -1.05560400 -1.64587100
H 7.37034400 0.65473300 -1.18194900
H 7.93974500 -0.57680100 -0.03622300
C 0.98154900 -1.11662200 0.26669500
C 1.70694300 0.09499300 0.54348600
C 0.89503300 1.27396300 0.66629800
C -0.45689800 1.24835100 0.55698800
C -1.17731700 0.01744700 0.31009300
C -0.37100100 -1.17602900 0.19838500
H -1.00427300 2.17850500 0.66469700
H -0.84984700 -2.13661900 0.04327100
B -2.69696300 -0.01270500 0.15554200
C -4.95551500 -3.04318900 -0.84645600
C -4.27628900 -1.82680300 -0.89600500
C -3.46930100 -1.40663900 0.19301600
C -3.39369900 -2.26143200 1.31784800
C -4.10134500 -3.46998400 1.34014900
C -4.88569900 -3.88298300 0.26814900
H -5.56220300 -3.34519200 -1.69831600
H -4.03839800 -4.10011100 2.22551900
C -3.48610100 1.35698600 -0.05116700
C -4.52338200 1.75395500 0.83186000
C -5.19879600 2.95862700 0.63602500
C -4.89852500 3.80916800 -0.43021500
C -3.89242000 3.41535400 -1.30679600
C -3.18517400 2.21977000 -1.13258200
H -5.98394800 3.24291100 1.33411700
H -3.64894900 4.05268900 -2.15512000
C -4.40117500 -0.98757700 -2.14782900
H -4.81433000 0.00102000 -1.93065000
H -3.42522800 -0.83086000 -2.61937200
H -5.04808400 -1.47354500 -2.88332900
C -4.90338500 0.90835800 2.02677400
H -5.15613000 -0.11378400 1.73400000
H -4.07698400 0.84175300 2.74294600
H -5.76059100 1.33671000 2.55308500
C -5.64242100 -5.18866100 0.29822700
H -5.28455400 -5.87288100 -0.47901400
H -5.53141100 -5.69227700 1.26215500
H -6.71179700 -5.03111300 0.12261600
C -2.56888600 -1.91872200 2.54152300
H -1.56528900 -2.35483000 2.47438500
H -2.43508500 -0.84251200 2.66396700
H -3.03742900 -2.31283200 3.44834000
C -5.63399400 5.11509500 -0.60784500
H -5.29673100 5.85894300 0.12308700
H -5.47236900 5.53452700 -1.60444400
H -6.71149700 4.98694900 -0.46504400
C -2.10962100 1.90556100 -2.15295300
H -1.15399100 2.36263800 -1.86994300
H -1.93035100 0.83317100 -2.24952300
H -2.38319600 2.29856500 -3.13687900
F 1.54610300 2.44601900 0.85007900
F 1.73450300 -2.23409100 0.14253600

Ac-B-CN

<u>*S_0*</u>

```
C 3.59312100 -1.83032700 3.15019400
C 2.92771700 -1.22018500 2.09294800
C 3.64803300 -0.68992900 1.01547900
C 5.04959900 -0.80410600 0.97097200
C 5.68902100 -1.39767100 2.06088200
C 4.98096300 -1.90668000 3.14712800
C 5.08800400 0.81670400 -0.91770800
C 3.68546600 0.90509500 -0.84572200
C 3.00078800 1.89956900 -1.55484800
H 1.91924600 1.95157800 -1.52141300
C 3.70240700 2.83894100 -2.30194900
C 5.09124600 2.79965400 -2.34055900
C 5.76398400 1.79111600 -1.65440400
H 3.01984800 -2.23209600 3.97883100
H 1.84694400 -1.15143000 2.11828800
H 6.76876000 -1.48524100 2.06123800
H 5.51062300 -2.36805800 3.97319500
H 3.15606700 3.60533900 -2.84134200
H 5.65005400 3.53449200 -2.90938600
H 6.84499800 1.75746600 -1.71523800
N 2.98455900 -0.03120300 -0.04803000
C 5.78894100 -0.39698300 -0.30474100
C 5.66856100 -1.57105300 -1.31540500
H 6.13670000 -2.47046600 -0.90315500
H 4.62418400 -1.80215200 -1.53634600
H 6.16697200 -1.31172500 -2.25471600
C 7.27938100 -0.13035600 -0.04390600
H 7.79821400 0.11332200 -0.97367900
H 7.42699500 0.68814400 0.66618600
H 7.76900100 -1.02513900 0.34696600
C 0.84961800 -0.92418000 -0.81150500
C 1.56812100 0.00228100 -0.04135200
C 0.84689700 0.93459700 0.72764800
C -0.55258000 0.93608000 0.70816800
C -1.27631600 -0.00025700 -0.04375800
C -0.55063300 -0.93142000 -0.79741500
H -1.08522400 1.67833300 1.29442200
H -1.07953800 -1.67768700 -1.38170900
B -2.85964100 -0.01469400 -0.03200100
C -5.18424000 -2.73279100 -1.53048400
C -4.57799100 -1.50108200 -1.28921700
C -3.58507700 -1.38727700 -0.28102500
C -3.26110800 -2.53815300 0.47582800
C -3.91071400 -3.74907600 0.22017000
C -4.86595300 -3.87209200 -0.78617000
H -5.93294200 -2.80736600 -2.31576000
H -3.66302800 -4.61802900 0.82570900
C -3.61595000 1.33773700 0.23069100
C -4.61094500 1.41950400 1.24065300
C -5.24385100 2.63500800 1.49509500
C -4.95183800 3.78872000 0.76254900
C -3.99652500 3.69674400 -0.24693100
C -3.32059800 2.50329900 -0.51595700
H -5.99336900 2.68496500 2.28144900
H -3.77002200 4.57691700 -0.84446700
C -4.99869700 -0.31543800 -2.13201000
H -5.50413400 0.44306100 -1.52797500
H -4.14484500 0.17359600 -2.61093700
H -5.68322800 -0.62821200 -2.92397800
C -5.00646000 0.21798900 2.07312300
H -5.47914300 -0.55341700 1.45920000
H -4.14436800 -0.24667100 2.56141600
H -5.71057900 0.50670400 2.85689600
C -5.53252600 -5.19278900 -1.07978600
H -5.11207100 -5.64606600 -1.98421900
```

H -5.39771500 -5.90294100 -0.26045000
H -6.60580600 -5.06590600 -1.24840700
C -2.24821300 -2.51921100 1.60440300
H -1.26157700 -2.84587000 1.25778500
H -2.12158100 -1.52993100 2.04924100
H -2.55424100 -3.20203300 2.40147500
C -5.64635400 5.09129300 1.07151500
H -5.23047200 5.54608300 1.97729800
H -5.53271000 5.81134900 0.25761800
H -6.71543900 4.93894400 1.24547900
C -2.31158800 2.51962300 -1.64818400
H -1.33098000 2.86267200 -1.30011200
H -2.16573200 1.53918000 -2.10619400
H -2.63451100 3.20644300 -2.43504200
C 1.54507700 1.89629700 1.53321400
N 2.07365700 2.68901100 2.19689000
C 1.55985200 -1.88605700 -1.60625200
N 2.09049900 -2.68259500 -2.26405900

Vertical S_1

C 3.59312 -1.83033 3.15019
C 2.92772 -1.22019 2.09295
C 3.64803 -0.68993 1.01548
C 5.0496 -0.80411 0.97097
C 5.68902 -1.39767 2.06088
C 4.98096 -1.90668 3.14713
C 5.088 0.8167 -0.91771
C 3.68547 0.9051 -0.84572
C 3.00079 1.89957 -1.55485
H 1.91925 1.95158 -1.52141
C 3.70241 2.83894 -2.30195
C 5.09125 2.79965 -2.34056
C 5.76398 1.79112 -1.6544
H 3.01985 -2.2321 3.97883
H 1.84694 -1.15143 2.11829
H 6.76876 -1.48524 2.06124
H 5.51062 -2.36806 3.9732
H 3.15607 3.60534 -2.84134
H 5.65005 3.53449 -2.90939
H 6.845 1.75747 -1.71524
N 2.98456 -0.0312 -0.04803
C 5.78894 -0.39698 -0.30474
C 5.66856 -1.57105 -1.31541
H 6.1367 -2.47047 -0.90316
H 4.62418 -1.80215 -1.53635
H 6.16697 -1.31173 -2.25472
C 7.27938 -0.13036 -0.04391
H 7.79821 0.11332 -0.97368
H 7.42699 0.68814 0.66619
H 7.769 -1.02514 0.34697
C 0.84962 -0.92418 -0.81151
C 1.56812 0.00228 -0.04135
C 0.8469 0.9346 0.72765
C -0.55258 0.93608 0.70817
C -1.27632 -0.00026 -0.04376
C -0.55063 -0.93142 -0.79742
H -1.08522 1.67833 1.29442
H -1.07954 -1.67769 -1.38171
B -2.85964 -0.01469 -0.032
C -5.18424 -2.73279 -1.53048
C -4.57799 -1.50108 -1.28922
C -3.58508 -1.38728 -0.28103
C -3.26111 -2.53815 0.47583
C -3.91071 -3.74908 0.22017
C -4.86595 -3.87209 -0.78617
H -5.93294 -2.80737 -2.31576
H -3.66303 -4.61803 0.82571
C -3.61595 1.33774 0.23069

```
C -4.61095 1.4195 1.24065
C -5.24385 2.63501 1.4951
C -4.95184 3.78872 0.76255
C -3.99653 3.69674 -0.24693
C -3.3206 2.5033 -0.51596
H -5.99337 2.68497 2.28145
H -3.77002 4.57692 -0.84447
C -4.9987 -0.31544 -2.13201
H -5.50413 0.44306 -1.52798
H -4.14485 0.1736 -2.61094
H -5.68323 -0.62821 -2.92398
C -5.00646 0.21799 2.07312
H -5.47914 -0.55342 1.4592
H -4.14437 -0.24667 2.56142
H -5.71058 0.5067 2.8569
C -5.53253 -5.19279 -1.07979
H -5.11207 -5.64607 -1.98422
H -5.39771 -5.90294 -0.26045
H -6.60581 -5.06591 -1.24841
C -2.24821 -2.51921 1.6044
H -1.26158 -2.84587 1.25779
H -2.12158 -1.52993 2.04924
H -2.55424 -3.20203 2.40148
C -5.64635 5.09129 1.07151
H -5.23047 5.54608 1.9773
H -5.53271 5.81135 0.25762
H -6.71544 4.93894 1.24548
C -2.31159 2.51962 -1.64818
H -1.33098 2.86267 -1.30011
H -2.16573 1.53918 -2.10619
H -2.63451 3.20644 -2.43504
C 1.54508 1.8963 1.53321
N 2.07366 2.68901 2.19689
C 1.55985 -1.88606 -1.60625
N 2.0905 -2.6826 -2.26406
```

Vertical T_1

```
C 3.59312 -1.83033 3.15019
C 2.92772 -1.22019 2.09295
C 3.64803 -0.68993 1.01548
C 5.0496 -0.80411 0.97097
C 5.68902 -1.39767 2.06088
C 4.98096 -1.90668 3.14713
C 5.088 0.8167 -0.91771
C 3.68547 0.9051 -0.84572
C 3.00079 1.89957 -1.55485
H 1.91925 1.95158 -1.52141
C 3.70241 2.83894 -2.30195
C 5.09125 2.79965 -2.34056
C 5.76398 1.79112 -1.6544
H 3.01985 -2.2321 3.97883
H 1.84694 -1.15143 2.11829
H 6.76876 -1.48524 2.06124
H 5.51062 -2.36806 3.9732
H 3.15607 3.60534 -2.84134
H 5.65005 3.53449 -2.90939
H 6.845 1.75747 -1.71524
N 2.98456 -0.0312 -0.04803
C 5.78894 -0.39698 -0.30474
C 5.66856 -1.57105 -1.31541
H 6.1367 -2.47047 -0.90316
H 4.62418 -1.80215 -1.53635
H 6.16697 -1.31173 -2.25472
C 7.27938 -0.13036 -0.04391
H 7.79821 0.11332 -0.97368
H 7.42699 0.68814 0.66619
H 7.769 -1.02514 0.34697
C 0.84962 -0.92418 -0.81151
C 1.56812 0.00228 -0.04135
```

C 0.8469 0.9346 0.72765
C -0.55258 0.93608 0.70817
C -1.27632 -0.00026 -0.04376
C -0.55063 -0.93142 -0.79742
H -1.08522 1.67833 1.29442
H -1.07954 -1.67769 -1.38171
B -2.85964 -0.01469 -0.032
C -5.18424 -2.73279 -1.53048
C -4.57799 -1.50108 -1.28922
C -3.58508 -1.38728 -0.28103
C -3.26111 -2.53815 0.47583
C -3.91071 -3.74908 0.22017
C -4.86595 -3.87209 -0.78617
H -5.93294 -2.80737 -2.31576
H -3.66303 -4.61803 0.82571
C -3.61595 1.33774 0.23069
C -4.61095 1.4195 1.24065
C -5.24385 2.63501 1.4951
C -4.95184 3.78872 0.76255
C -3.99653 3.69674 -0.24693
C -3.3206 2.5033 -0.51596
H -5.99337 2.68497 2.28145
H -3.77002 4.57692 -0.84447
C -4.9987 -0.31544 -2.13201
H -5.50413 0.44306 -1.52798
H -4.14485 0.1736 -2.61094
H -5.68323 -0.62821 -2.92398
C -5.00646 0.21799 2.07312
H -5.47914 -0.55342 1.4592
H -4.14437 -0.24667 2.56142
H -5.71058 0.5067 2.8569
C -5.53253 -5.19279 -1.07979
H -5.11207 -5.64607 -1.98422
H -5.39771 -5.90294 -0.26045
H -6.60581 -5.06591 -1.24841
C -2.24821 -2.51921 1.6044
H -1.26158 -2.84587 1.25779
H -2.12158 -1.52993 2.04924
H -2.55424 -3.20203 2.40148
C -5.64635 5.09129 1.07151
H -5.23047 5.54608 1.9773
H -5.53271 5.81135 0.25762
H -6.71544 4.93894 1.24548
C -2.31159 2.51962 -1.64818
H -1.33098 2.86267 -1.30011
H -2.16573 1.53918 -2.10619
H -2.63451 3.20644 -2.43504
C 1.54508 1.8963 1.53321
N 2.07366 2.68901 2.19689
C 1.55985 -1.88606 -1.60625
N 2.0905 -2.6826 -2.26406

Adiabatic S_1

C 3.15937400 -1.35996500 3.31367400
C 2.65840900 -0.82950800 2.14250000
C 3.53968600 -0.41230900 1.11619000
C 4.94127800 -0.53501700 1.27816000
C 5.40696200 -1.07988800 2.47232900
C 4.53989700 -1.48914200 3.48244500
C 5.21567600 0.48402900 -1.00586400
C 3.80503700 0.56898400 -1.08758300
C 3.17995000 1.11813900 -2.23146400
H 2.10062700 1.16255500 -2.25758700
C 3.94276200 1.58092100 -3.28358400
C 5.33623800 1.49955200 -3.21587100
C 5.95100600 0.95563900 -2.09050800
H 2.47695900 -1.67523300 4.09343900
H 1.59318100 -0.72710100 2.00144500
H 6.47387200 -1.19706400 2.62416300

H 4.94252300 -1.90926500 4.39701300
H 3.45833200 2.00249500 -4.15576300
H 5.94499900 1.85656200 -4.03872800
H 7.03329300 0.89653300 -2.06567800
N 3.00240200 0.11352300 -0.05178900
C 5.92412700 -0.11913500 0.19550400
C 6.70002600 -1.38094100 -0.27375700
H 7.23602500 -1.83524900 0.56292300
H 6.01364800 -2.12235400 -0.68917200
H 7.43298800 -1.11768500 -1.03986700
C 6.90665000 0.92985000 0.78067700
H 7.64419900 1.22893700 0.03297700
H 6.36853900 1.82099800 1.11163000
H 7.45050500 0.51794000 1.63338400
C 0.87515900 -0.98995800 -0.68870800
C 1.57865600 0.16324600 -0.20478700
C 0.82600400 1.27153400 0.30943500
C -0.55570000 1.21294400 0.31186000
C -1.28556600 0.07801900 -0.15612400
C -0.50607600 -1.00692600 -0.66346400
H -1.10567700 2.06217100 0.70531300
H -1.01789400 -1.88159300 -1.05261600
B -2.81624600 0.01189000 -0.08534300
C -5.15099600 -2.78170300 -1.50681600
C -4.52366000 -1.55036600 -1.32086300
C -3.54761400 -1.38673900 -0.30433400
C -3.25752000 -2.50571600 0.51161100
C -3.91763100 -3.72353000 0.30568800
C -4.86201600 -3.88708000 -0.70260300
H -5.89091800 -2.88274200 -2.29836500
H -3.68448400 -4.56414500 0.95652400
C -3.64808100 1.33445600 0.22378700
C -4.51012600 1.40659500 1.34886300
C -5.23030300 2.57049700 1.61321400
C -5.14883000 3.69398900 0.78633800
C -4.31410200 3.62017800 -0.32367800
C -3.56303700 2.47292600 -0.61058800
H -5.87872100 2.60373800 2.48656800
H -4.24174500 4.47608000 -0.99209200
C -4.89688400 -0.40977400 -2.24176200
H -5.31306600 0.43501000 -1.68644500
H -4.02526400 -0.03547700 -2.78838800
H -5.63580000 -0.72926300 -2.98120100
C -4.65013900 0.24587000 2.30860800
H -4.97541100 -0.66317800 1.79628300
H -3.69650100 0.01722600 2.79655200
H -5.37451800 0.47276600 3.09518800
C -5.54458200 -5.21252400 -0.93799500
H -5.14719400 -5.70385000 -1.83327800
H -5.40025600 -5.89388900 -0.09549000
H -6.62072800 -5.08384500 -1.08961800
C -2.25108000 -2.45587900 1.64466600
H -1.27612600 -2.84076200 1.32241300
H -2.08615300 -1.44242000 2.01331500
H -2.58544500 -3.07458500 2.48283900
C -5.93508400 4.94262300 1.10410300
H -5.55351900 5.42611100 2.01020600
H -5.87816700 5.67070200 0.29076100
H -6.99139600 4.71472300 1.27922200
C -2.68332000 2.51838500 -1.84460200
H -1.73326000 3.02291900 -1.63245000
H -2.43530500 1.52488600 -2.22069900
H -3.17491500 3.07445100 -2.64863600
C 1.51474000 2.41358700 0.81612100
N 2.12334800 3.32053500 1.22277900
C 1.61793600 -2.10161900 -1.18792400
N 2.27577200 -2.98434400 -1.57052300

Adiabatic T_1

C 3.00262500 -1.36763400 3.27628700

C 2.55715700 -0.82162800 2.08991900
C 3.48499000 -0.41116300 1.10297200
C 4.87642100 -0.55771600 1.31891500
C 5.28629500 -1.11689600 2.52701700
C 4.37337700 -1.51890700 3.49877600
C 5.25427900 0.46890600 -0.94644900
C 3.84937900 0.58306700 -1.08122600
C 3.28208600 1.15587300 -2.24384700
H 2.20724600 1.23073200 -2.31781800
C 4.09573800 1.60917600 -3.26179300
C 5.48311800 1.49540800 -3.14268300
C 6.04127200 0.93132900 -1.99829400
H 2.28539400 -1.67768900 4.02623900
H 1.50067000 -0.70017700 1.90309800
H 6.34476400 -1.25095400 2.71943600
H 4.73288700 -1.95079700 4.42576000
H 3.65382900 2.04842100 -4.14774900
H 6.13119900 1.84406700 -3.93864400
H 7.12005700 0.84840800 -1.93055300
N 3.00023300 0.13185000 -0.08111600
C 5.90622800 -0.15202200 0.27767100
C 6.68071400 -1.42334600 -0.16765600
H 7.18060900 -1.88690000 0.68614400
H 5.99908800 -2.15407100 -0.60901200
H 7.44367800 -1.16919700 -0.90713300
C 6.88100500 0.87931600 0.90562400
H 7.65191500 1.16966700 0.18872100
H 6.34401900 1.77759100 1.21873100
H 7.38391300 0.45548800 1.77743100
C 0.87977900 -0.95715600 -0.75866600
C 1.58332800 0.20174100 -0.28550300
C 0.82723500 1.30282900 0.24245500
C -0.55392200 1.23510100 0.26003700
C -1.28133400 0.09617600 -0.20027700
C -0.50072800 -0.98312800 -0.71775100
H -1.10510500 2.08065700 0.65979900
H -1.01132900 -1.86047800 -1.10242600
B -2.81045200 0.01808600 -0.10619300
C -5.14056400 -2.79053900 -1.50450200
C -4.51863900 -1.55561300 -1.32507500
C -3.53478200 -1.38558700 -0.31704800
C -3.23071500 -2.50218200 0.49657200
C -3.88593500 -3.72390400 0.29780800
C -4.83844300 -3.89354500 -0.70167100
H -5.88653000 -2.89654200 -2.28967800
H -3.64199100 -4.56252700 0.94730500
C -3.64780500 1.33341200 0.21849900
C -4.49193700 1.39680900 1.35756200
C -5.21745400 2.55429000 1.63535600
C -5.15933700 3.67914600 0.80839900
C -4.34193400 3.61382600 -0.31502200
C -3.58528700 2.47369200 -0.61537900
H -5.85098100 2.58120900 2.51979800
H -4.28752400 4.47121000 -0.98321800
C -4.90533900 -0.41806200 -2.24400900
H -5.31702000 0.42708600 -1.68591000
H -4.04090900 -0.04345400 -2.80174900
H -5.65189200 -0.74075800 -2.97432500
C -4.60707400 0.23340000 2.31738800
H -4.94074900 -0.67548400 1.81022900
H -3.64266900 0.00643900 2.78440600
H -5.31527900 0.45610600 3.11978100
C -5.51549400 -5.22313400 -0.92954900
H -5.11807100 -5.71653100 -1.82367700
H -5.36568100 -5.90005600 -0.08441100
H -6.59258500 -5.10007600 -1.07887800
C -2.21490200 -2.44661400 1.62108600
H -1.25136100 -2.86084900 1.30123600
H -2.02670300 -1.42878800 1.96517400
H -2.55613500 -3.03811000 2.47614500

C -5.95228800 4.92012500 1.13927400
H -5.56949300 5.39971200 2.04688700
H -5.90444200 5.65416700 0.33076100
H -7.00619800 4.68383200 1.31792200
C -2.72376100 2.52902900 -1.86171700
H -1.77157600 3.03374600 -1.66005000
H -2.47933400 1.53883500 -2.24859500
H -3.22792300 3.08981400 -2.65458200
C 1.51371200 2.44691700 0.74747700
N 2.12026600 3.35489800 1.15498000
C 1.62397400 -2.06585800 -1.26261900
N 2.28296500 -2.94728700 -1.64611800

Ac-B-NO_2

S_0

C -3.76552900 0.24966700 3.63806100
C -3.01800300 0.18296000 2.46820500
C -3.65291700 0.14137000 1.22098000
C -5.05445200 0.23563800 1.13471100
C -5.77631900 0.27921800 2.32934500
C -5.15337200 0.27667000 3.57454100
C -4.91623900 -0.30709800 -1.29959200
C -3.52244800 -0.42549100 -1.16463400
C -2.75550000 -0.98689800 -2.19309500
H -1.68373500 -1.09328600 -2.07957400
C -3.36164500 -1.43593100 -3.35908900
C -4.74171200 -1.34704200 -3.50203100
C -5.49772600 -0.79022400 -2.47445200
H -3.25432400 0.27248700 4.59439000
H -1.93717200 0.16396000 2.53973900
H -6.85740100 0.33656200 2.29384400
H -5.74815600 0.31492800 4.48044800
H -2.75047900 -1.86621000 -4.14526300
H -5.22847700 -1.70208300 -4.40362100
H -6.57038400 -0.71574800 -2.60554000
N -2.90502500 0.01682700 0.02940800
C -5.71923400 0.43429900 -0.22970100
C -5.66819700 1.95004600 -0.56409600
H -6.21563100 2.52129200 0.19251100
H -4.63972200 2.31684300 -0.58900800
H -6.12323000 2.13338600 -1.54284700
C -7.19161400 -0.01059300 -0.21552300
H -7.66495700 0.17888800 -1.18120200
H -7.29154200 -1.07381300 0.01930900
H -7.76551800 0.56569400 0.51335900
C -0.75781500 1.07367300 -0.43721500
C -1.49481800 0.00412200 0.08412200
C -0.73273400 -1.05418500 0.60450100
C 0.65571500 -1.03999500 0.61069100
C 1.36990600 0.02748800 0.05045900
C 0.62841000 1.08905900 -0.48387000
H 1.18291500 -1.88492400 1.04018800
H 1.13279400 1.94309600 -0.92339000
B 2.95258800 0.03496000 0.01809200
C 5.25014900 2.94148700 -1.12021100
C 4.63865000 1.69131300 -1.05276200
C 3.68566900 1.42454000 -0.03424100
C 3.41004200 2.44008000 0.91068900
C 4.06582800 3.67182800 0.82506900
C 4.97938700 3.94827400 -0.18895900
H 5.96642500 3.13706900 -1.91485400
H 3.85612500 4.43261200 1.57360700
C 3.70345200 -1.34577800 0.03456300
C 4.70293800 -1.60131300 1.00995100
C 5.33822900 -2.84107800 1.04363600
C 5.04574000 -3.84695100 0.11836600

C 4.08368600 -3.58162500 -0.85314700
C 3.40285900 -2.36175700 -0.90223100
H 6.09085400 -3.02837100 1.80600100
H 3.85510300 -4.34207600 -1.59642600
C 5.00617700 0.65440200 -2.09254500
H 5.50573600 -0.20546400 -1.63818600
H 4.12691100 0.27136700 -2.61960800
H 5.67754000 1.07867600 -2.84286400
C 5.09764400 -0.56263500 2.03789800
H 5.57012300 0.30384700 1.56726800
H 4.23437500 -0.19091300 2.59851100
H 5.80134800 -0.98062700 2.76170300
C 5.65194800 5.29413000 -0.29498100
H 5.21917900 5.88086700 -1.11254400
H 5.53858300 5.87153800 0.62563800
H 6.72099600 5.18706100 -0.50089700
C 2.44471600 2.24774000 2.06455600
H 1.47112000 2.70174400 1.84927000
H 2.26514000 1.19772400 2.30288900
H 2.83200200 2.72723000 2.96783000
C 5.74624300 -5.18101500 0.18452700
H 5.33299700 -5.79743400 0.99036700
H 5.63513700 -5.73733300 -0.74936500
H 6.81480300 -5.05787900 0.38316900
C 2.38213200 -2.18060800 -2.00935800
H 1.42187100 -2.63546200 -1.74188500
H 2.18817000 -1.13272200 -2.24646900
H 2.72514200 -2.66660600 -2.92686200
N -1.38327300 -2.25129900 1.16496100
O -2.33228200 -2.72191000 0.55978000
O -0.89282800 -2.70530400 2.19132700
N -1.45742800 2.25835400 -0.95644500
O -2.25082600 2.81195400 -0.21046600
O -1.15318800 2.62593300 -2.08243700

Vertical S_1

C -3.76553 0.24967 3.63806
C -3.018 0.18296 2.46821
C -3.65292 0.14137 1.22098
C -5.05445 0.23564 1.13471
C -5.77632 0.27922 2.32934
C -5.15337 0.27667 3.57454
C -4.91624 -0.3071 -1.29959
C -3.52245 -0.42549 -1.16463
C -2.7555 -0.9869 -2.1931
H -1.68374 -1.09329 -2.07957
C -3.36165 -1.43593 -3.35909
C -4.74171 -1.34704 -3.50203
C -5.49773 -0.79022 -2.47445
H -3.25432 0.27249 4.59439
H -1.93717 0.16396 2.53974
H -6.8574 0.33656 2.29384
H -5.74816 0.31493 4.48045
H -2.75048 -1.86621 -4.14526
H -5.22848 -1.70208 -4.40362
H -6.57038 -0.71575 -2.60554
N -2.90503 0.01683 0.02941
C -5.71923 0.4343 -0.2297
C -5.6682 1.95005 -0.5641
H -6.21563 2.52129 0.19251
H -4.63972 2.31684 -0.58901
H -6.12323 2.13339 -1.54285
C -7.19161 -0.01059 -0.21552
H -7.66496 0.17889 -1.1812
H -7.29154 -1.07381 0.01931
H -7.76552 0.56569 0.51336
C -0.75782 1.07367 -0.43722

C -1.49482 0.00412 0.08412
C -0.73273 -1.05418 0.6045
C 0.65572 -1.04 0.61069
C 1.36991 0.02749 0.05046
C 0.62841 1.08906 -0.48387
H 1.18292 -1.88492 1.04019
H 1.13279 1.9431 -0.92339
B 2.95259 0.03496 0.01809
C 5.25015 2.94149 -1.12021
C 4.63865 1.69131 -1.05276
C 3.68567 1.42454 -0.03424
C 3.41004 2.44008 0.91069
C 4.06583 3.67183 0.82507
C 4.97939 3.94827 -0.18896
H 5.96643 3.13707 -1.91485
H 3.85613 4.43261 1.57361
C 3.70345 -1.34578 0.03456
C 4.70294 -1.60131 1.00995
C 5.33823 -2.84108 1.04364
C 5.04574 -3.84695 0.11837
C 4.08369 -3.58163 -0.85315
C 3.40286 -2.36176 -0.90223
H 6.09085 -3.02837 1.806
H 3.8551 -4.34208 -1.59643
C 5.00618 0.6544 -2.09254
H 5.50574 -0.20546 -1.63819
H 4.12691 0.27137 -2.61961
H 5.67754 1.07868 -2.84286
C 5.09764 -0.56264 2.0379
H 5.57012 0.30385 1.56727
H 4.23438 -0.19091 2.59851
H 5.80135 -0.98063 2.7617
C 5.65195 5.29413 -0.29498
H 5.21918 5.88087 -1.11254
H 5.53858 5.87154 0.62564
H 6.721 5.18706 -0.5009
C 2.44472 2.24774 2.06456
H 1.47112 2.70174 1.84927
H 2.26514 1.19772 2.30289
H 2.832 2.72723 2.96783
C 5.74624 -5.18102 0.18453
H 5.333 -5.79743 0.99037
H 5.63514 -5.73733 -0.74937
H 6.8148 -5.05788 0.38317
C 2.38213 -2.18061 -2.00936
H 1.42187 -2.63546 -1.74189
H 2.18817 -1.13272 -2.24647
H 2.72514 -2.66661 -2.92686
N -1.38327 -2.2513 1.16496
O -2.33228 -2.72191 0.55978
O -0.89283 -2.7053 2.19133
N -1.45743 2.25835 -0.95645
O -2.25083 2.81195 -0.21047
O -1.15319 2.62593 -2.08244

Vertical T_1

C -3.76553 0.24967 3.63806
C -3.018 0.18296 2.46821
C -3.65292 0.14137 1.22098
C -5.05445 0.23564 1.13471
C -5.77632 0.27922 2.32934
C -5.15337 0.27667 3.57454
C -4.91624 -0.3071 -1.29959
C -3.52245 -0.42549 -1.16463
C -2.7555 -0.9869 -2.1931
H -1.68374 -1.09329 -2.07957
C -3.36165 -1.43593 -3.35909

C -4.74171 -1.34704 -3.50203
C -5.49773 -0.79022 -2.47445
H -3.25432 0.27249 4.59439
H -1.93717 0.16396 2.53974
H -6.8574 0.33656 2.29384
H -5.74816 0.31493 4.48045
H -2.75048 -1.86621 -4.14526
H -5.22848 -1.70208 -4.40362
H -6.57038 -0.71575 -2.60554
N -2.90503 0.01683 0.02941
C -5.71923 0.4343 -0.2297
C -5.6682 1.95005 -0.5641
H -6.21563 2.52129 0.19251
H -4.63972 2.31684 -0.58901
H -6.12323 2.13339 -1.54285
C -7.19161 -0.01059 -0.21552
H -7.66496 0.17889 -1.1812
H -7.29154 -1.07381 0.01931
H -7.76552 0.56569 0.51336
C -0.75782 1.07367 -0.43722
C -1.49482 0.00412 0.08412
C -0.73273 -1.05418 0.6045
C 0.65572 -1.04 0.61069
C 1.36991 0.02749 0.05046
C 0.62841 1.08906 -0.48387
H 1.18292 -1.88492 1.04019
H 1.13279 1.9431 -0.92339
B 2.95259 0.03496 0.01809
C 5.25015 2.94149 -1.12021
C 4.63865 1.69131 -1.05276
C 3.68567 1.42454 -0.03424
C 3.41004 2.44008 0.91069
C 4.06583 3.67183 0.82507
C 4.97939 3.94827 -0.18896
H 5.96643 3.13707 -1.91485
H 3.85613 4.43261 1.57361
C 3.70345 -1.34578 0.03456
C 4.70294 -1.60131 1.00995
C 5.33823 -2.84108 1.04364
C 5.04574 -3.84695 0.11837
C 4.08369 -3.58163 -0.85315
C 3.40286 -2.36176 -0.90223
H 6.09085 -3.02837 1.806
H 3.8551 -4.34208 -1.59643
C 5.00618 0.6544 -2.09254
H 5.50574 -0.20546 -1.63819
H 4.12691 0.27137 -2.61961
H 5.67754 1.07868 -2.84286
C 5.09764 -0.56264 2.0379
H 5.57012 0.30385 1.56727
H 4.23438 -0.19091 2.59851
H 5.80135 -0.98063 2.7617
C 5.65195 5.29413 -0.29498
H 5.21918 5.88087 -1.11254
H 5.53858 5.87154 0.62564
H 6.721 5.18706 -0.5009
C 2.44472 2.24774 2.06456
H 1.47112 2.70174 1.84927
H 2.26514 1.19772 2.30289
H 2.832 2.72723 2.96783
C 5.74624 -5.18102 0.18453
H 5.333 -5.79743 0.99037
H 5.63514 -5.73733 -0.74937
H 6.8148 -5.05788 0.38317
C 2.38213 -2.18061 -2.00936
H 1.42187 -2.63546 -1.74189
H 2.18817 -1.13272 -2.24647
H 2.72514 -2.66661 -2.92686
N -1.38327 -2.2513 1.16496
O -2.33228 -2.72191 0.55978

O -0.89283 -2.7053 2.19133
N -1.45743 2.25835 -0.95645
O -2.25083 2.81195 -0.21047
O -1.15319 2.62593 -2.08244

Adiabatic S_1

C -3.44721000 1.81065700 3.16347100
C -2.81541100 1.26151800 2.06785300
C -3.57025400 0.64260500 1.04666100
C -4.97883300 0.58169700 1.13152100
C -5.58182300 1.13897200 2.25749700
C -4.83940800 1.74710500 3.26481700
C -4.98703000 -0.56921100 -1.10430300
C -3.57697600 -0.48369700 -1.10472500
C -2.82832200 -0.98353700 -2.19397000
H -1.74985800 -0.92792200 -2.16848200
C -3.46834000 -1.55051300 -3.27656500
C -4.86252300 -1.63801500 -3.28925200
C -5.59804300 -1.15518200 -2.21147000
H -2.86003700 2.28230300 3.94217700
H -1.73964700 1.30049100 1.98595000
H -6.65982100 1.09122800 2.36196600
H -5.34436800 2.16810100 4.12677000
H -2.88511000 -1.93014500 -4.10679600
H -5.37430000 -2.08711200 -4.13278400
H -6.67773800 -1.24991100 -2.23408400
N -2.91086400 0.09152900 -0.03677000
C -5.82603200 -0.10119300 0.07196300
C -6.90706300 0.88829700 -0.43302800
H -7.54762300 1.21852600 0.38748000
H -6.44395200 1.76826300 -0.88556300
H -7.55208700 0.41544600 -1.17668200
C -6.48838900 -1.35126700 0.71637700
H -7.11940400 -1.86753900 -0.01170100
H -5.72405800 -2.04428400 1.07250100
H -7.11735600 -1.05679600 1.56047900
C -0.72031200 1.06335400 -0.67134900
C -1.47225900 0.06311400 -0.02506600
C -0.78493800 -1.00814200 0.61177900
C 0.62581700 -0.98901000 0.58870100
C 1.36252600 0.00851400 -0.03576900
C 0.65934500 1.05174000 -0.69153200
H 1.11705100 -1.81852600 1.08402000
H 1.18842000 1.84520100 -1.20350800
B 2.93522800 -0.01740000 0.00022900
C 5.35675700 2.73880600 -1.28200500
C 4.70396900 1.51450600 -1.14213400
C 3.70875300 1.35149000 -0.14525100
C 3.42754500 2.44626500 0.70447100
C 4.12260300 3.64956700 0.55134500
C 5.08211700 3.82183600 -0.44341100
H 6.10765500 2.85055500 -2.06122800
H 3.90587000 4.47290800 1.22867500
C 3.67506000 -1.39808100 0.17246300
C 4.65513800 -1.57154400 1.18440800
C 5.28045200 -2.80658000 1.34560400
C 4.99355500 -3.89325100 0.51549300
C 4.05405800 -3.71098900 -0.49541300
C 3.38668300 -2.49534500 -0.67257700
H 6.01688900 -2.92627000 2.13726700
H 3.82778300 -4.53741600 -1.16557700
C 5.08128500 0.38743200 -2.07982500
H 5.56400800 -0.43293400 -1.54144500
H 4.20818700 -0.03504000 -2.58639200
H 5.77072000 0.73934800 -2.85132700
C 5.04124600 -0.44690800 2.12129100
H 5.53543300 0.36799500 1.58509200

```
H 4.17046100 -0.01661300 2.62507200
H 5.72342900 -0.80588500 2.89589700
C 5.79367000 5.13933100 -0.62853400
H 5.38558300 5.68393600 -1.48720300
H 5.68773900 5.77945700 0.25089200
H 6.86152000 4.99124600 -0.81486300
C 2.40049000 2.37164800 1.81704900
H 1.42026500 2.71508200 1.46770200
H 2.26349500 1.36008300 2.20495600
H 2.69432800 3.01205400 2.65339800
C 5.67194600 -5.22375400 0.72747000
H 5.24771500 -5.73968300 1.59580800
H 5.55116900 -5.87876400 -0.13890900
H 6.74281800 -5.09833100 0.91376900
C 2.38632200 -2.41970300 -1.80977900
H 1.40557100 -2.78879100 -1.49115200
H 2.24159700 -1.40520600 -2.18657300
H 2.71584200 -3.04178500 -2.64699800
N -1.42965500 -2.08542300 1.23776300
O -2.69714800 -2.17703800 1.14513800
O -0.72177000 -2.93251600 1.85442900
N -1.38070500 2.18222500 -1.36032600
O -2.46137700 2.57644700 -0.92888400
O -0.80505400 2.67063600 -2.32319100
```

Adiabatic T_1

```
C -3.37785400 1.72364800 3.21396400
C -2.77114300 1.21737400 2.08372100
C -3.54671100 0.61754500 1.06733500
C -4.95115100 0.53877000 1.18795100
C -5.52811200 1.04925600 2.34951200
C -4.76494400 1.63463900 3.35457300
C -5.01245000 -0.50312100 -1.09941700
C -3.60357800 -0.40978600 -1.13515200
C -2.88546000 -0.84152300 -2.27225100
H -1.80668300 -0.78906400 -2.28157700
C -3.55550300 -1.34292200 -3.36981800
C -4.94845500 -1.43791500 -3.34783400
C -5.65322900 -1.02685000 -2.22128200
H -2.77435300 2.18222000 3.98802400
H -1.69930600 1.27995200 1.97113000
H -6.60143400 0.98040200 2.48443800
H -5.25033500 2.02020300 4.24394000
H -2.99487300 -1.66770000 -4.23809800
H -5.48285900 -1.83930900 -4.20131800
H -6.73204100 -1.13157400 -2.21606400
N -2.91042800 0.10096300 -0.04867600
C -5.81757400 -0.12660500 0.13285400
C -6.96494800 0.83260000 -0.27136200
H -7.57607800 1.09820700 0.59359300
H -6.56454200 1.75124100 -0.70671900
H -7.62934100 0.36491400 -1.00076000
C -6.39167500 -1.43701100 0.74296400
H -7.03326900 -1.94543100 0.01835400
H -5.58102000 -2.10958200 1.02909900
H -6.99008400 -1.21169500 1.62978500
C -0.71674600 1.06991200 -0.68534400
C -1.47276000 0.06043000 -0.05929900
C -0.78783200 -1.02502800 0.55284000
C 0.62234300 -1.00894100 0.54006900
C 1.36276200 0.00033100 -0.06101900
C 0.66307600 1.05492700 -0.70219400
H 1.10977800 -1.84805500 1.02299600
H 1.19557700 1.85788200 -1.19569200
B 2.93536600 -0.02478200 -0.01064000
C 5.37120200 2.74288900 -1.23795600
C 4.71365900 1.51874300 -1.11996000
C 3.71082300 1.34516200 -0.13263700
```

C 3.42576200 2.42918500 0.72940700
C 4.12610100 3.63200200 0.59870200
C 5.09413300 3.81472600 -0.38599200
H 6.12808500 2.86313400 -2.01008400
H 3.90684900 4.44645800 1.28587200
C 3.67354400 -1.40708900 0.15456200
C 4.64157400 -1.59212300 1.17624400
C 5.26385600 -2.82929800 1.33163500
C 4.98601300 -3.90689100 0.48649700
C 4.05895700 -3.71299600 -0.53349200
C 3.39480800 -2.49473000 -0.70579900
H 5.99083600 -2.95816400 2.13057200
H 3.84016900 -4.53174000 -1.21546900
C 5.09415200 0.40345300 -2.07027100
H 5.57342300 -0.42425500 -1.54007200
H 4.22297300 -0.01152200 -2.58622200
H 5.78761400 0.76418700 -2.83406400
C 5.01646000 -0.47826900 2.13034100
H 5.51173200 0.34522100 1.60851600
H 4.14014800 -0.05755400 2.63273900
H 5.69377900 -0.84468000 2.90572800
C 5.81180400 5.13222400 -0.54589700
H 5.40303300 5.69774800 -1.39062300
H 5.71264700 5.75330100 0.34789000
H 6.87809100 4.98276100 -0.73965500
C 2.38833200 2.34225000 1.83144000
H 1.41302800 2.69545200 1.47797600
H 2.24346700 1.32535400 2.20219000
H 2.67695100 2.96823700 2.68046200
C 5.66145300 -5.23986600 0.69233900
H 5.23479700 -5.75980000 1.55706400
H 5.54116700 -5.88964800 -0.17799200
H 6.73223800 -5.11735200 0.88127400
C 2.40947200 -2.40630800 -1.85516000
H 1.43098500 -2.80107500 -1.56127400
H 2.25182300 -1.38498500 -2.20704400
H 2.76204100 -2.99988800 -2.70365900
N -1.44289100 -2.11429000 1.14243600
O -2.71223300 -2.18701000 1.03680800
O -0.75091900 -2.98823400 1.73861100
N -1.37370800 2.20833800 -1.34398100
O -2.44069000 2.60888400 -0.88555800
O -0.80786200 2.70489500 -2.30853600

Figure S1. UV-Vis. spectra of the studied complexes.

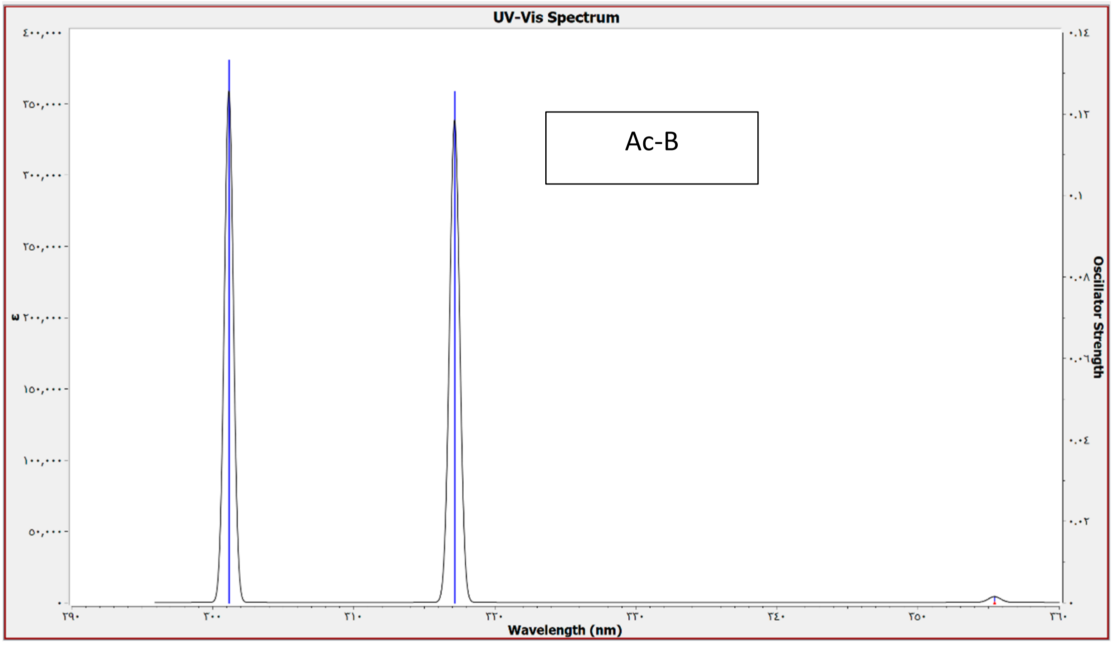

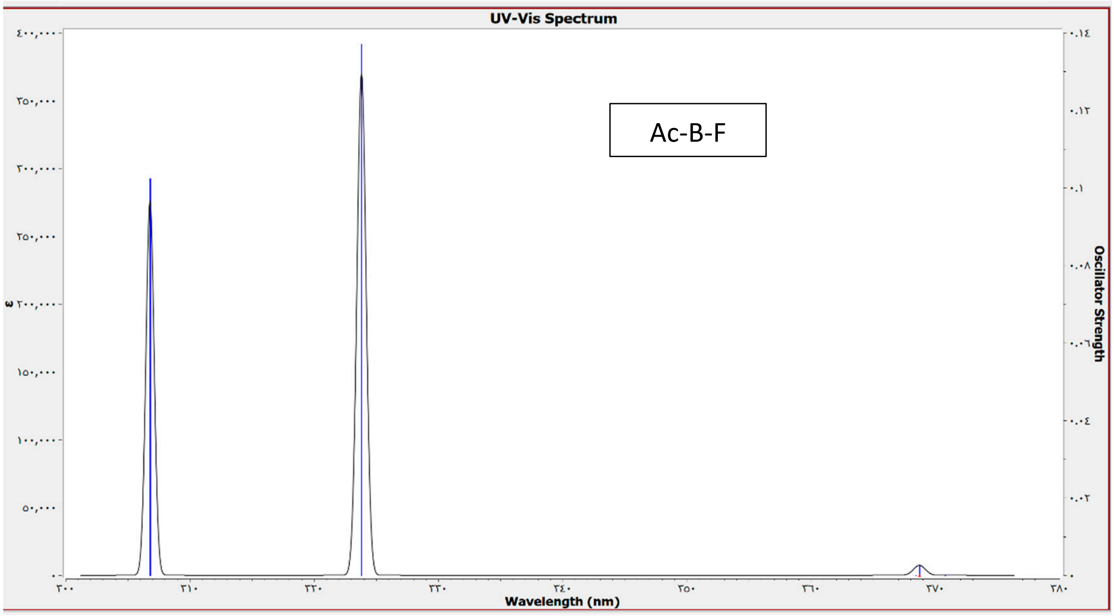
UV-Vis Spectrum
Ac-B-F
ε
Oscillator Strength
Wavelength (nm)

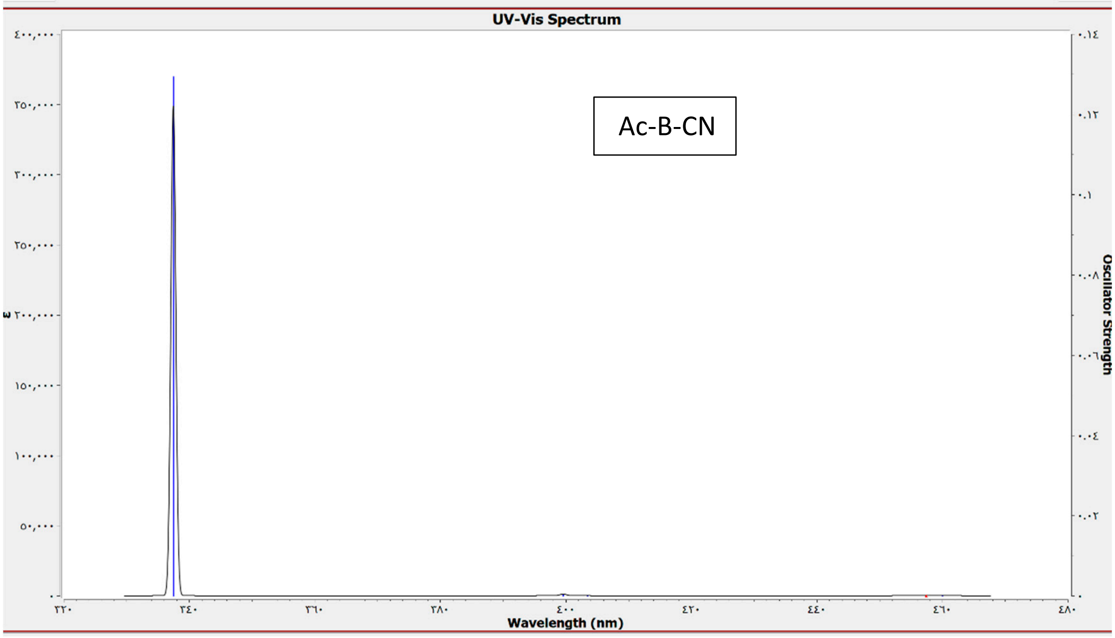
UV-Vis Spectrum
Ac-B-CN
ε
Oscillator Strength
Wavelength (nm)

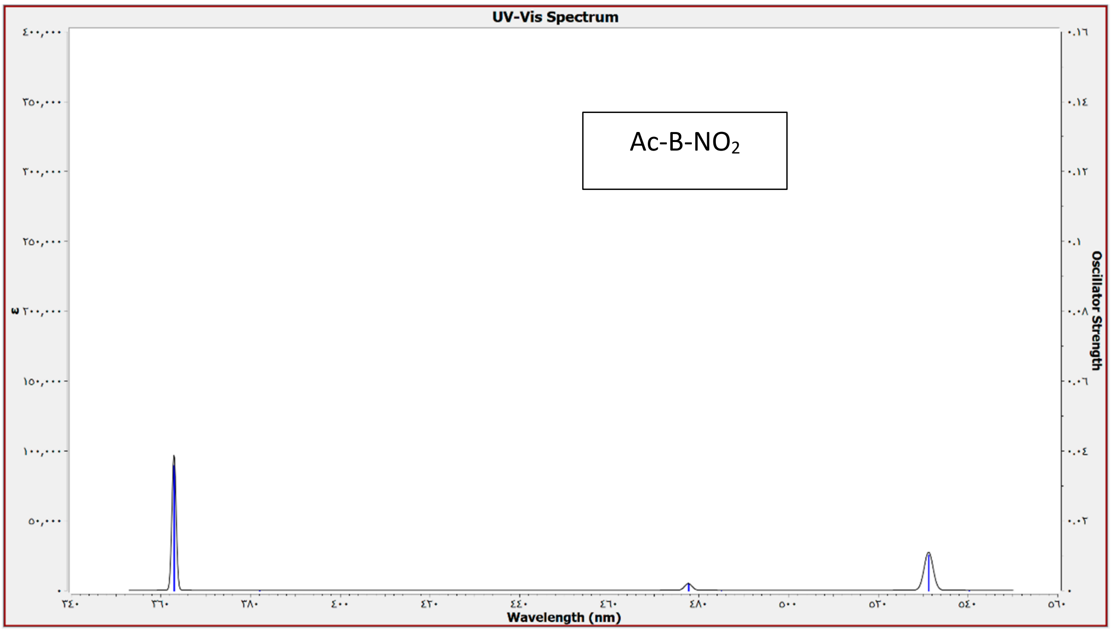

Figure S2. NTO of the second excited triplet state (T_2).

Compound	□ole	Electron
**Ac-B**	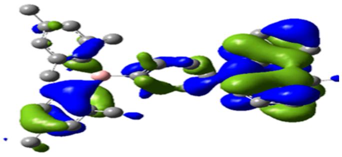	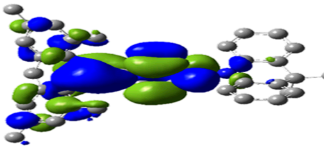
**Ac-B-F**	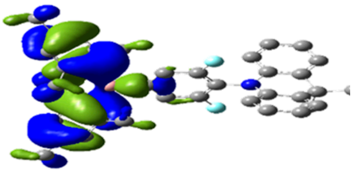	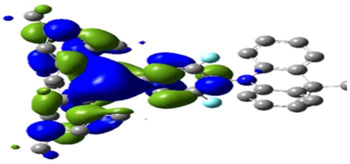
**Ac-B-CN**	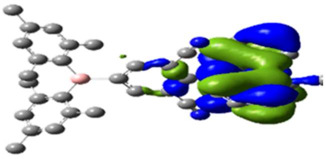	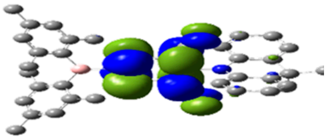
**Ac-B-NO$_2$**	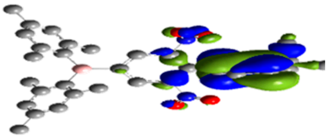	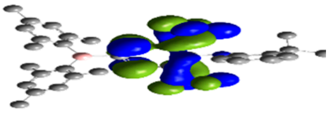

Figure S3. NTO of the third excited triplet state (T_3).

Compound	Hole	Electron
**Ac-B**	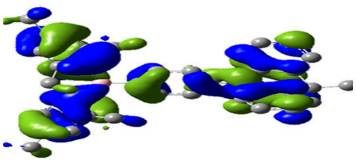	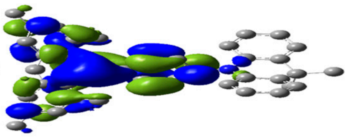
**Ac-B-F**	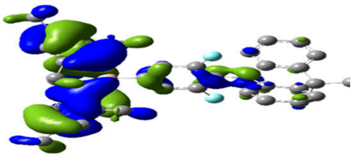	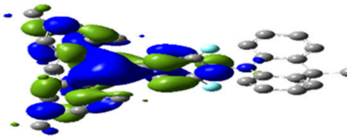
**Ac-B-CN**	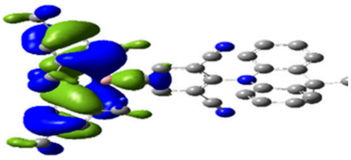	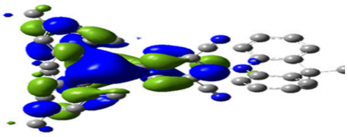
**Ac-B-NO_2**	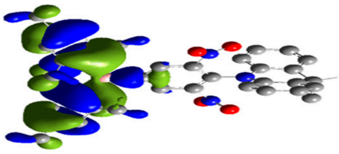	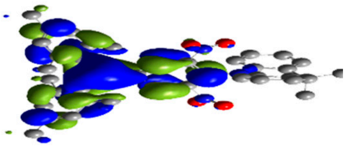

References

1. Helfrich, W.; Schneider, W. Recombination Radiation in Anthracene Crystals. *Phys. Rev. Lett.* **1956,** *14*, 229.
2. Murawski, C.; Gather, M.C. Emerging Biomedical Applications of Organic Light-Emitting Diodes. *Adv. Opt. Mater.* **2021,** *9*, 2100269.
3. Tang, C.W.; VanSlyke, S.A. Organic electroluminescent diodes. *Appl. Phys. Lett.* **1987**, *51*, 913–915.
4. Zhao, D.; Qin, Z.; Huang, J.; Yu, J. Progress on material, structure and function for tandem organic light-emitting diodes. *Org. Electron.* **2017**, *51*, 220–242.
5. Lichtman, J.W.; Conchello, J.-A. Fluorescence microscopy. *Nat. Methods* **2005**, *2*, 910–919.
6. Baldo, M.A.; O'Brien, D.F.; You, Y.; Shoustikov, A.; Sibley, S.; Thompson, M.E.; Forrest, S.R. Highly efficient phosphorescent emission from organic electroluminescent devices. *Nature* **1998**, *395*, 151–154.
7. Yersin, H. Triplet Emitters for OLED Applications. Mechanisms of Exciton Trapping and Control of Emission Properties. *Transit. Met. Rare Earth Compd.* **2004**, *Vol. 241*, 1–26.
8. Chang, C.F.; Cheng, Y.M.; Chi, Y.; Chiu, Y.C.; Lin, C.C.; Lee, G.H.; Chou, P.T.; Chen, C.C.; Chang, C.H.; Wu, C.C. Highly Efficient Blue-Emitting Iridium(III) Carbene Complexes and Phosphorescent OLEDs. *Angew. Chem. Int. Ed.* **2008**, *47*, 4542–4545.
9. Uoyama, H.; Goushi, K.; Shizu, K.; Nomura, H.; Adachi, C. Highly efficient organic light-emitting diodes from delayed fluorescence. *Nature* **2012**, *492*, 234–238.
10. Endo, A.; Sato, K.; Yoshimura, K.; Kai, T.; Kawada, A.; Miyazaki, H.; Adachi, C. Efficient up-conversion of triplet excitons into a singlet state and its application for organic light emitting diodes. *Appl. Phys. Lett.* **2011**, *98*, 42.
11. Zhang, D.-W.; Li, M.; Chen, C.-F. Recent advances in circularly polarized electroluminescence based on organic light-emitting diodes. *Chem. Soc. Rev.* **2020**, *49*, 1331–1343.
12. Kawasumi, K.; Wu, T.; Zhu, T.; Chae, H.S.; Van Voorhis, T.; Baldo, M.A.; Swager, T.M. Thermally Activated Delayed Fluorescence Materials Based on Homoconjugation Effect of Donor–Acceptor Triptycenes. *J. Am. Chem. Soc.* **2015**, *137*, 11908–11911.

13. Hirata, S.; Sakai, Y.; Masui, K.; Tanaka, H.; Lee, S.Y.; Nomura, H.; Nakamura, N.; Yasumatsu, M.; Nakanotani, H.; Zhang, Q. Highly efficient blue electroluminescence based on thermally activated delayed fluorescence. *Nat. Mater.* **2015**, *14*, 330–336.
14. Nakanotani, H.; Higuchi, T.; Furukawa, T.; Masui, K.; Morimoto, K.; Numata, M.; Tanaka, H.; Sagara, Y.; Yasuda, T.; Adachi, C. High-efficiency organic light-emitting diodes with fluorescent emitters. *Nat Commun* **2014**, *5*, 4016.
15. Chen, X.-K.; Zhang, S.-F.; Fan, J.-X.; Ren, A.-M. Nature of Highly Efficient Thermally Activated Delayed Fluorescence in Organic Light-Emitting Diode Emitters: Nonadiabatic Effect between Excited States. *J. Phys. Chem. C* **2015**, *119*, 9728–9733.
16. Huang, S.; Zhang, Q.; Shiota, Y.; Nakagawa, T.; Kuwabara, K.; Yoshizawa, K.; Adachi, C. Computational Prediction for Singlet- and Triplet-Transition Energies of Charge-Transfer Compounds. *J. Chem. Theory Comput.* **2013**, *9*, 3872–3877.
17. Sun, H.; Zhong, C.; Bredas, J.-L. Reliable Prediction with Tuned Range-Separated Functionals of the Singlet–Triplet Gap in Organic Emitters for Thermally Activated Delayed Fluorescence. *J. Chem. Theory Comput.* **2015**, *11*, 3851–3858.
18. Naveen, K.R.; Lee, H.; Braveenth, R.; Karthik, D.; Yang, K.J.; Hwang, S.J.; Kwon, J.H. Achieving High Efficiency and Pure Blue Color in Hyperfluorescence Organic Light Emitting Diodes using Organo-Boron Based Emitters. *Adv. Funct. Mater.* **2022**, *32*, 2110356–2110366.
19. Dias, F.B.; Bourdakos, K.N.; Jankus, V.; Moss, K.C.; Kamtekar, K.T.; Bhalla Santos, J.; Bryce, M.R.; Monkman, A.P. Triplet Harvesting with 100% Efficiency by Way of Thermally Activated Delayed Fluorescence in Charge Transfer OLED Emitters. *Adv. Mater.* **2013**, *25*, 3707–3714.
20. Kim, J.U.; Park, I.S.; Chan, C.-Y.; Tanaka, M.; Tsuchiya, Y.; Nakanotani, H.; Adachi, C. Nanosecond-time-scale delayed fluorescence molecule for deep-blue OLEDs with small efficiency rolloff. *Nat. Commun.* **2020**, *11*, 1765.
21. Etherington, M.K.; Gibson, J.; Higginbotham, H.F.; Penfold, T.J.; Monkman, A.P. Revealing the spin–vibronic coupling mechanism of thermally activated delayed fluorescence. *Nature Commun.* **2016**, *7*, 13680.
22. Monkman, A.P. Vibrational coupling in TADF and how molecular structure can control this complex triplet harvesting process (Conference Presentation). In *Organic Light Emitting Materials and Devices XXI;* SPIE: Paris, France, 2017; p. 1036204.
23. Fan, J.; Cai, L.; Lin, L.; Wang, C. Understanding the light-emitting mechanism of an X-shape organic thermally activated delayed fluorescence molecule: First-principles study. *Chem. Phys. Lett.* **2016**, *664*, 33–38.
24. Sagara, Y.; Shizu, K.; Tanaka, H.; Miyazaki, H.; Goushi, K.; Kaji, H.; Adachi, C. Highly Efficient Thermally Activated Delayed Fluorescence Emitters with a Small Singlet Triplet Energy Gap and Large Oscillator Strength. *Chem. Lett.* **2015**, *44*, 360–362.
25. Ansari, R.; Shao, W.; Yoon, S.-J.; Kim, J.; Kieffer, J. Charge Transfer as the Key Parameter Affecting the Color Purity of Thermally Activated Delayed Fluorescence Emitters. *ACS Appl. Mater. Interfaces* **2021**, *13*, 28529–28537.
26. Pan, K.C.; Li, S.W.; Ho, Y.Y.; Shiu, Y.J.; Tsai, W.L.; Jiao, M.; Lee, W.K.; Wu, C.C.; Chung, C.L.; Chatterjee, T. Efficient and Tunable Thermally Activated Delayed Fluorescence Emitters Having Orientation-Adjustable CN-Substituted Pyridine and Pyrimidine Acceptor Units. *Adv. Funct. Mater.* **2016**, *26*, 7560–7571.
27. Zhao, B.; Wang, H.; Han, C.; Ma, P.; Li, Z.; Chang, P.; Xu, H. Highly Efficient Deep-Red Non-Doped Diodes Based on a T-Shape Thermally Activated Delayed Fluorescence Emitter. *Angew. Chem. Int. Ed.* **2020**, *59*, 19042–19047.
28. Ward, J.S.; Nobuyasu, R.S.; Fox, M.A.; Aguilar, J.A.; Hall, D.; Batsanov, A.S.; Ren, Z.; Dias, F.B.; Bryce, M.R. Impact of Methoxy Substituents on Thermally Activated Delayed Fluorescence and Room-Temperature Phosphorescence in All-Organic Donor–Acceptor Systems. *J. Org. Chem.* **2019**, *84*, 3801–3816.
29. Kitamoto, Y.; Namikawa, T.; Suzuki, T.; Miyata, Y.; Kita, H.; Sato, T.; Oi, S. Dimesitylarylborane-based luminescent emitters exhibiting highly-efficient thermally activated delayed fluorescence for organic light-emitting diodes. *Org. Electron.* **2016**, *34*, 208–217.
30. Shizu, K.; Tanaka, H.; Uejima, M.; Sato, T.; Tanaka, K.; Kaji, H.; Adachi, C. Strategy for Designing Electron Donors for Thermally Activated Delayed Fluorescence Emitters. *J. Phys. Chem.* **2015**, *C 119*, 1291-1297.
31. Ganesan, P.; Ranganathan, R.; Chi, Y.; Liu, X.K.; Lee, C.S.; Liu, S.H.; Lee, G.H.; Lin, T.C.; Y. T.; Chen, Y.T.; Chou, P.T. Functional Pyrimidine-Based Thermally Activated Delay Fluorescence Emitters: Photophysics, Mechanochromism, and Fabrication of Organic Light-Emitting Diodes. *Chem. A Eur. J.* **2017**, *23*, 2858–2866.
32. Cho, E.; Liu, L.; Coropceanu, V.; Brédas, J.-L. Impact of secondary donor units on the excited-state properties and thermally activated delayed fluorescence (TADF) efficiency of pentacarbazole-benzonitrile emitters. *J. Chem. Phys.* **2020**, *153*, 144708.
33. Li, J.; Chen, W.-C.; Liu, H.; Chen, Z.; Chai, D.; Lee, C.-S.; Yang, C. Double-twist pyridine–carbonitrile derivatives yielding excellent thermally activated delayed fluorescence emitters for high-performance OLEDs. *J. Mater. Chem.* **2020**, **C** *8*, 602-606.
34. Lv, X.; Huang, R.; Sun, S.; Zhang, Q.; Xiang, S.; Ye, S.; Leng, P.; Dias, F.B.; Wang, L. Blue TADF Emitters Based on Indenocarbazole Derivatives with High Photoluminescence and Electroluminescence Efficiencies. *ACS Appl. Mater. Interfaces* **2019**, *11*, 10758–10767.
35. Kumar, A.; Lee, W.; Lee, T.; Jung, J.; Yoo, S.; Lee, M.H. Triarylboron-based TADF emitters with perfluoro substituents: High-efficiency OLEDs with a power efficiency over 100 lm W^{-1}. *J. Mater. Chem.* **2020,** *C8*, 4253-4263.
36. Marcus, R.A. Electron Transfer Reactions in Chemistry: Theory and Experiment (Nobel Lecture). *Angew. Chem. Int. Ed. Engl.* **1993,** *32*, 1111–1121.
37. Hilborn, R.C. Einstein coefficients, cross sections, f values, dipole moments, and all that. *Am. J. Phys.* **1982,** *50*, 982–986.
38. Thangaraji, V.; Rajamalli, P.; Jayakumar, J.; Huang, M.-J.; Chen, Y.-W.; Cheng, C.-H. Quinolinylmethanone-Based Thermally Activated Delayed Fluorescence Emitters and the Application in OLEDs: Effect of Intramolecular H-Bonding. *ACS Appl. Mater. Interfaces* **2019**, *11*, 17128–17133.
39. Olivier, Y.; Sancho-Garcia, J.-C.; Muccioli, L.; D'Avino, G.; Beljonne, D. Computational Design of Thermally Activated Delayed Fluorescence Materials: The Challenges Ahead. *J. Phys. Chem. Lett.* **2018**, *9*, 6149–6163.

40. De Silva, P.; Kim, C.A.; Zhu, T.; Van Voorhis, T. Extracting Design Principles for Efficient Thermally Activated Delayed Fluorescence (TADF) from a Simple Four-State Model. *Chem. Mater.* **2019**, *31*, 6995–7006.
41. Samanta, P.K.; Kim, D.; Coropceanu, V.; Brédas, J.-L. Up-Conversion Intersystem Crossing Rates in Organic Emitters for Thermally Activated Delayed Fluorescence: Impact of the Nature of Singlet vs Triplet Excited States. *J. Am. Chem. Soc.* **2017**, *139*, 4042–4051.
42. Yang, Z.; Mao, Z.; Xie, Z.; Zhang, Y.; Liu, S.; Zhao, J.; Xu, J.; Chi, Z.; Aldred, M.P. Recent advances in organic thermally activated delayed fluorescence materials. *Chem. Soc. Rev.* **2017**, *46*, 915–1016.
43. Yang, C.Y.; Lee, K.H.; Lee, J.Y. Zig-Zag Type Molecular Design Strategy of N-Type Hosts for Sky-Blue Thermally-Activated Delayed Fluorescence Organic Light-Emitting Diodes. *Chem. A Eur. J.* **2020**, *26*, 2429–2435.
44. Hussain, A.; Yuan, H.; Li, W.; Zhang, J. Theoretical investigations of the realization of sky-blue to blue TADF materials via CH/N and H/CN substitution at the diphenylsulphone acceptor. *J. Mater. Chem.* **2019**, *C 7*, 6685-6691.
45. Chen, X.K.; Tsuchiya, Y.; Ishikawa, Y.; Zhong, C.; Adachi, C.; Brédas, J.-L. A New Design Strategy for Efficient Thermally Activated Delayed Fluorescence Organic Emitters: From Twisted to Planar Structures. *Adv. Mater.* **2017**, *29*, 1702767.
46. Meng, G.; Chen, X.; Wang, X.; Wang, N.; Peng, T.; Wang, S. Isomeric Bright Sky-Blue TADF Emitters Based on Bisacridine Decorated DBNA: Impact of Donor Locations on Luminescent and Electroluminescent Properties. *Adv. Opt. Mater.* **2019**, *7*, 1900130.
47. ADF 2013.01, SCM, Theoretical Chemistry. Vrije Universiteit, Amsterdam, The Netherlands. Available online: http://www.scm.com.
48. Zhang, Q.; Li, J.; Shizu, K.; Huang, S.; Hirata, S.; Miyazaki, H.; Adachi, C. Design of Efficient Thermally Activated Delayed Fluorescence Materials for Pure Blue Organic Light Emitting Diodes. *J. Am. Chem. Soc.* **2012**, *134*, 14706–14709.
49. Chen, M.C.; Chen, D.G.; Chou, P.T. Fluorescent Chromophores Containing the Nitro Group: Relatively Unexplored Emissive Properties. *ChemPlusChem* **2021**, *86*, 11–27.
50. Sun, J.; Zhang, J.; Liang, Q.; Wei, Y.; Duan, C.; Han, C.; Xu, H. Charge-Transfer Exciton Manipulation Based on Hydrogen Bond for Efficient White Thermally Activated Delayed Fluorescence. *Adv. Funct. Mater.* **2020**, *30*, 1908568.
51. Espinosa, E.; Molins, E.; Lecomte, C. Hydrogen bond strengths revealed by topological analyses of experimentally observed electron densities. *Chem. Phys. Lett.* **1998**, *285*, 170.
52. Gupta, A.K.; Zhang, Z.; Spuling, E.; Kaczmarek, M.; Wang, Y.; Hassan, Z.; Samuel, I.D.; Bräse, S.; Zysman-Colman, E. Electron-withdrawing group modified carbazolophane donors for deep blue thermally activated delayed fluorescence OLEDs. *Mater. Adv.* **2021**, *2*, 6684–6693.
53. Yi, C.-L.; Ko, C.-L.; Yeh, T.-C.; Chen, C.-Y.; Chen, Y.-S.; Chen, D.-G.; Chou, P.-T.; Hung, W.-Y.; Wong, K.-T. Harnessing a New Co-Host System and Low Concentration of New TADF Emitters Equipped with Trifluoromethyl- and Cyano-Substituted Benzene as Core for High-Efficiency Blue OLEDs. *ACS Appl. Mater. Interfaces* **2019**, *12*, 2724–2732.
54. Woon, K.L.; Nadiah, Z.N.; Hasan, Z.A.; Ariffin, A.; Chen, S.-A. Tuning the singlet-triplet energy splitting by fluorination at 3,6 positions of the 1,4-biscarbazoylbenzene. *Dye. Pigment.* **2016**, *132*, 1–6.
55. Lu, T.; Chen, F. Multiwfn: A Multifunctional Wavefunction Analyzer. *J. Comput. Chem.* **2012**, *33*, 580–592.
56. Frisch, M.J.; Trucks, G.W.; Schlegel, H.B.; Scuseria, G.E.; Robb, M.A.; Cheeseman, J.R.; Scalmani, G.; Barone, V.; Mennucci, B.; Petersson, G.A.; et. al. *Gaussian 09*; Gaussian, Inc.: Wallingford, CT, USA, 2009.
57. Chai, J.-D.; Head-Gordon, M. Long-range corrected hybrid density functionals with damped atom-atom dispersion corrections. *Phys. Chem. Chem. Phys.* **2008**, *10*, 6615–6620.
58. Ditchfield, R.; Hehre, W.J.; Pople, J.A. Self-Consistent Molecular Orbital Methods. 9. Extended Gaussian-type basis for molecular-orbital studies of organic molecules. *J. Chem. Phys.* **1971**, *54*, 724.
59. Bauernschmitt, R.; Ahlrichs, R. Treatment of electronic excitations within the adiabatic approximation of time dependent density functional theory. *Chem. Phys. Lett.* **1996**, *256*, 454–464.
60. Martin, R.L. Natural transition orbitals. *J. Chem. Phys.* **2003**, *118*, 4775–4777.
61. Bader, R.F. A quantum theory of molecular structure and its applications. *Chem. Rev.* **1991**, *91*, 893–928.
62. Humphrey, W.; Dalke, A.; Schulten, K. VMD: Visual molecular dynamics. *J. Mol. Graph.* **1996**, *14*, 33–38.
63. Silvi, B.; Savin, A. Classification of chemical bonds based on topological analysis of electron localization functions. *Nature* **1994**, *371*, 683–686.
64. Lasorne, B.; Worth, G.A.; Robb, M.A. Excited-state dynamics. *Wiley Interdiscip. Rev. Comput. Mol. Sci. Wiley Online Libr.* **2011**, *1*, 460–475.
65. Mignolet, B.; Curchod, B.F.E. Excited-State Molecular Dynamics Triggered by Light Pulses—*Ab Initio* Multiple Spawning vs Trajectory Surface Hopping. *J. Phys. Chem. A* **2019**, *123*, *16*, 3582–3591.

Disclaimer/Publisher's Note: The statements, opinions and data contained in all publications are solely those of the individual author(s) and contributor(s) and not of MDPI and/or the editor(s). MDPI and/or the editor(s) disclaim responsibility for any injury to people or property resulting from any ideas, methods, instructions or products referred to in the content.
